# Biofilm-associated toxin and extracellular protease cooperatively suppress competitors in *Bacillus subtilis* biofilms

**DOI:** 10.1371/journal.pgen.1008232

**Published:** 2019-10-17

**Authors:** Kazuo Kobayashi, Yukako Ikemoto

**Affiliations:** Division of Biological Science, Nara Institute of Science & Technology, Ikoma, Nara, Japan; Danmarks Tekniske Universitet, DENMARK

## Abstract

In nature, most bacteria live in biofilms where they compete with their siblings and other species for space and nutrients. Some bacteria produce antibiotics in biofilms; however, since the diffusion of antibiotics is generally hindered in biofilms by extracellular polymeric substances, i.e., the biofilm matrix, their function remains unclear. The *Bacillus subtilis yitPOM* operon is a paralog of the *sdpABC* operon, which produces the secreted peptide toxin SDP. Unlike *sdpABC*, *yitPOM* is induced in biofilms by the DegS-DegU two-component regulatory system. High *yitPOM* expression leads to the production of a secreted toxin called YIT. Expression of *yitQ*, which lies upstream of *yitPOM*, confers resistance to the YIT toxin, suggesting that YitQ is an anti-toxin protein for the YIT toxin. The alternative sigma factor SigW also contributes to YIT toxin resistance. In a mutant lacking *yitQ* and *sigW*, the YIT toxin specifically inhibits biofilm formation, and the extracellular neutral protease NprB is required for this inhibition. The requirement for NprB is eliminated by Δ*eps* and Δ*bslA* mutations, either of which impairs production of biofilm matrix polymers. Overexpression of biofilm matrix polymers prevents the action of the SDP toxin but not the YIT toxin. These results indicate that, unlike the SDP toxin and many conventional antibiotics, the YIT toxin can pass through layers of biofilm matrix polymers to attack cells within biofilms with assistance from NprB. When the wild-type strain and the YIT-sensitive mutant were grown together on a solid medium, the wild-type strain formed biofilms that excluded the YIT-sensitive mutant. This observation suggests that the YIT toxin protects *B*. *subtilis* biofilms against competitors. Several bacteria are known to produce antibiotics in biofilms. We propose that some bacteria including *B*. *subtilis* may have evolved specialized antibiotics that can function within biofilms.

## Author summary

Biofilms are multicellular aggregates of bacteria that are formed on various living and non-living surfaces. Biofilms often cause serious problems, including food contamination and infectious diseases. Since bacteria in biofilms exhibit increased tolerance or resistance to antimicrobials, new agents and treatments for combating biofilm-related problems are required. In this study, we demonstrated that *B*. *subtilis* produces a secreted peptide antibiotic called the YIT toxin and its resistant proteins in biofilms. A mutant lacking the resistance genes was defective in biofilm formation. This effect resulted from the ability of the YIT toxin to pass through the biofilm defense barrier and to attack biofilm cells. Thus, unlike many conventional antibiotics, the YIT toxin can penetrate biofilms and suppress the growth of YIT toxin-sensitive cells within biofilms. Some bacteria produce antibiotics in biofilms, some of which can alter the bacterial composition in the biofilms. Taking these observations into consideration, our findings suggest that some bacteria produce special antibiotics that are effective against bacteria in biofilms, and these antibiotics might serve as anti-biofilm agents.

## Introduction

In the environment, bacteria compete for space and nutrients [1]. Antibiotics are thought to play a critical role in this competition, and antibiotic-producing bacteria are indeed common in various environments [2–4]. However, in the environment, most bacteria are found in sessile multicellular bacterial communities known as biofilms, in which bacteria exhibit increased antibiotic tolerance or resistance [5, 6]. Though alternative environmental roles of antibiotics have been proposed [7], this paradox has not been explained in detail to date.

In biofilms, bacterial cells adhere to each other and to a surface via a mixture of extracellular polymeric substances called the biofilm matrix, which consists of exopolysaccharides, proteins, nucleic acids, and/or lipids [8, 9]. When encased in the biofilm matrix, cells exhibit increased tolerance or resistance to environmental stresses, antibiotics, host defense systems, and predation [8, 9]. Thus, biofilm formation enables bacteria to remain in a favorable niche and to claim territory; however, biofilms are not a utopia for bacteria. The properties of biofilms, including high cell density, decreased internal fluidity, and, in many cases, the presence of multiple species, lead to conditions of harsh competition, especially when nutrients are scarce. Many bacteria secrete biofilm formation-inhibiting molecules, such as biosurfactants, polysaccharides, and molecules that interfere with bacterial quorum sensing, and these secreted molecules help to exclude unfavorable competitors from biofilms [10]. Antibiotics might also play an important role in competition within biofilms. However, since the properties of biofilms, including the protection of member cells by the biofilm matrix, the increased expression of antibiotic resistance genes, and the decreased internal fluidity, reduce the efficacy of antibiotics against biofilm cells [11–16], little attention has been paid to the functions of antibiotics in competition within biofilms. However, some biofilms do indeed produce antibiotics, and several of these antibiotics can alter the bacterial composition of the biofilm [17–23]. These observations suggest that the functions of antibiotics produced in biofilms remain to be investigated. An understanding of how bacteria use antibiotics in biofilms will not only provide insight into bacterial survival strategies within biofilms, it will also lead to the discovery of tactics for combating biofilm-related problems, such as food and beverage safety issues, industrial contamination, and biofilm-related diseases.

The Gram-positive soil bacterium *Bacillus subtilis* is a model organism for biofilm formation. *B*. *subtilis* forms robust biofilms under laboratory conditions, for example, pellicles on the surface of liquid media under static culture conditions or wrinkled colonies on solid media [24]. *B*. *subtilis* biofilms are maintained by a biofilm matrix that mainly consists of exopolysaccharides, TasA amyloid fibers, and BslA hydrophobins, which are produced by proteins encoded by the *epsABCDEFGHIJKLMNO* operon, the *tapA-sipW-tasA* operon, and *bslA*, respectively [24–29]. These genes are directly or indirectly repressed by the transcriptional repressors AbrB and SinR [30–33]. Phosphorylation of the response regulator Spo0A induces mechanisms that antagonize these repressors, leading to the expression of the biofilm matrix synthesis genes [34, 35].

*B*. *subtilis* produces a wide array of antibiotics. Many of these antibiotics are non-ribosomally synthesized peptide compounds, such as surfactin, bacillaene, fengycin, iturin, and bacilysin, which are thought to be important in nature for competition with other organisms, including fungi [4, 36]. Furthermore, *B*. *subtilis* produces ribosomally synthesized peptide antibiotics, such as bacteriocins and other protein-derived toxins, which are generally effective against other bacteria that are genetically similar and present in similar ecological niches [4, 37–39]. One of these protein-derived toxins is the cannibalism toxin SDP [40], whose function is involved in biofilm formation. The SDP toxin is derived from the internal sequence of SdpC, and it is encoded by the *sdpABC* operon. SdpC is a 203 amino acid protein that contains a typical N-terminal secretion signal and a C-terminal hydrophobic domain. After secretion and cleavage of the signal sequence, SdpC is further processed into the 42 amino acid peptide known as the SDP toxin, which corresponds to the C-terminal hydrophobic domain (C141 to S182) [39–41]. SdpA and SdpB are required for the processing of SdpC to SDP, and this processing is essential for the activity of the SDP toxin [41]. The hydrophobic nature of the SDP toxin enables the SDP toxin to penetrate bacterial membranes, where it then induces cell lysis by collapsing the proton motive force [42]. Downstream of *sdpABC* is the *sdpRI* operon, which encodes its own transcriptional repressor and an anti-toxin protein to the SDP toxin [43]. SdpI is an integral membrane protein that protects cells probably by binding to the SDP toxin. Transcription of the *sdpABC* and *sdpRI* operons is directly or indirectly activated by phosphorylated Spo0A [40, 43]. Spo0A is a master regulator of stationary phase development that is phosphorylated after the onset of stationary phase [44]. However, as the phosphorylation of Spo0A is subject to a bistable regulatory mechanism, a subset of *B*. *subtilis* cells produce the SDP toxin and the SdpI anti-toxin protein [40, 43]. Consequently, the secreted SDP toxin lyses and kills a fraction of the sibling cells that do not produce the SdpI anti-toxin protein. Since phosphorylated Spo0A also induces biofilm formation in parallel, cells that produce the SDP toxin and the SdpI anti-toxin protein efficiently develop biofilms by using nutrients released from their lysed siblings [45]. Moreover, the SDP toxin is effective not only against *B*. *subtilis*, but also against many Firmicutes bacteria [39, 41, 46]. Thus, the SDP toxin likely plays an important role in the early phase of biofilm formation by eliminating unnecessary types of cells and closely related competitors in the environment.

The undomesticated *B*. *subtilis* strain NCIB3610 encodes an *sdpABC* paralog known as *yitPOM*. While transcription of *sdpABC* is activated by Spo0A [40], *yitPOM* was previously identified as a member of the group of genes regulated by the DegS-DegU two-component regulatory system [47]. Phosphorylated DegU directly induces transcription of genes for the biofilm matrix protein, BslA, several antibiotic synthetases, and many extracellular degradative enzymes, such as proteases, levansucrase, α-amylase, β-glucanases, and xylanase. These antibiotics and degradative enzymes are thought to play important roles in bacterial competitions and nutrient acquisition in nature [47, 48, and references therein]. Since DegS-DegU is required for biofilm formation, we were interested in determining whether the *yitPOM*-encoded toxin plays a role particularly in biofilms. In this paper, we demonstrate that *yitPOM* encodes a biofilm-associated secreted toxin. Unlike many conventional antibiotics, in particular positive charged antibiotics, this toxin was able to attack cells within biofilms by passing through the layers of the biofilm matrix polymers with the assistance of an extracellular protease. Given that several other bacteria produce antibiotics in biofilms, our results suggest that some bacteria may have evolved specialized antibiotics to suppress competitors in biofilms.

## Results

### *yitPOM* encodes a toxin

**Fig 1:**
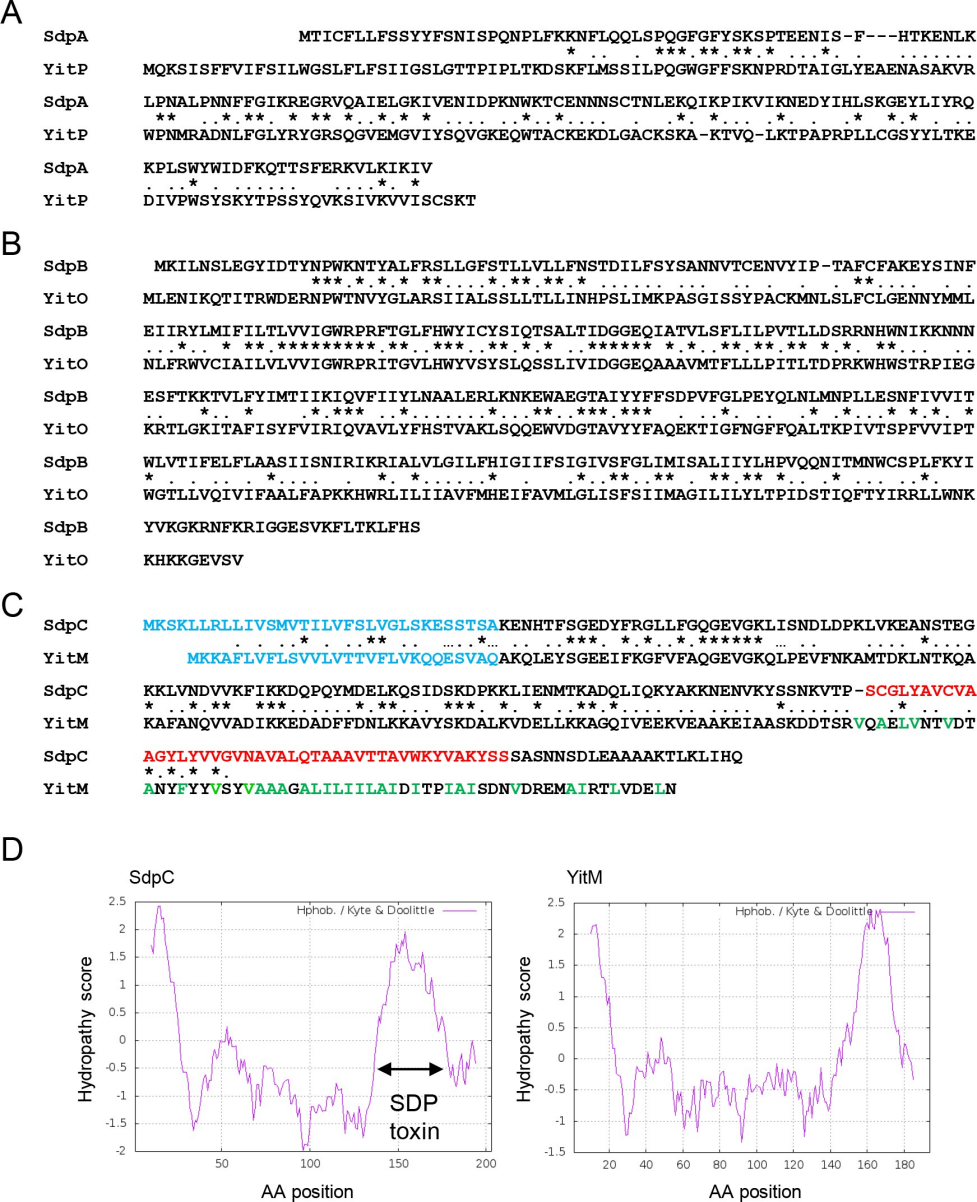
*yitPOM* is a paralog of *sdpABC*. (A) Alignment of SdpA and YitP. (B) Alignment of SdpB and YitO. (C) Alignment of SdpC and YitM. Identical and similar amino acid residues shared by the two proteins are indicated by asterisks and dots, respectively. The signal sequences of SdpC and YitM and the SDP toxin sequence are shown in blue and red, respectively. Hydrophobic amino acid residues in the C-terminal region of YitM are shown in green. (D) Kyte and Doolittle hydropathy plots of SdpC and YitM. The hydropathy score, representing the hydrophobic or hydrophilic properties of amino acid residues was calculated and plotted using the ExPASy website (https://web.expasy.org/protscale/) with a window size of 19.

The undomesticated *B*. *subtilis* strain NCIB3610 (hereafter referred to as the wild-type strain or 3610) [24] encodes an *sdpABC* paralog known as *yitPOM*. YitP and YitO exhibit approximately 50% sequence similarity to the entire SdpA and SdpB sequences, respectively (Fig 1A and 1B). Like SdpC, YitM has an N-terminal secretion signal; however, the sequence similarity between YitM and SdpC is limited to the N-terminal three quarters of the sequence, which does not include the entire sequence corresponding to the SDP toxin (Fig 1C). Although there is no sequence similarity, like SdpC, the YitM C-terminal region contains a hydrophobic domain (Fig 1C and 1D). These observations suggest the possibility that the C-terminal hydrophobic domain of YitM might be processed to a secreted toxin via a YitP and YitO-dependent mechanism. If this is the case, then *yitPOM* encodes a toxin whose sequence differs from that of the SDP toxin.

**Fig 2:**
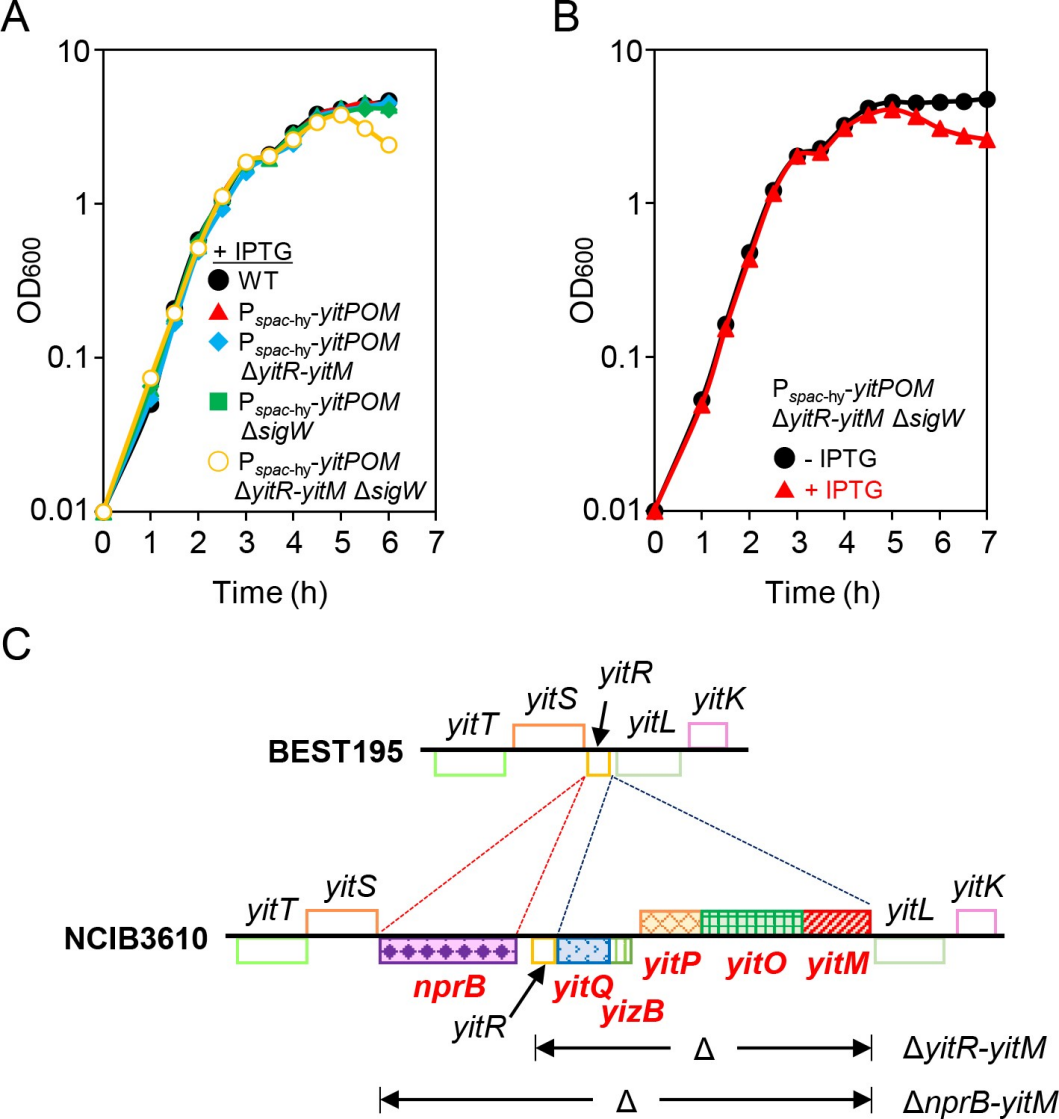
*yitPOM* encodes a toxin. (A) Effect of *yitPOM* induction on cell growth. *B*. *subtilis* strains were grown at 37°C in 2×SG supplemented with 1 mM IPTG with vigorous shaking. Growth profiles were examined at least three time, and the typical examples were shown. (B) Induction of *yitPOM* caused mild cell lysis. The P_*spac*-hy_-*yitPOM* Δ*yitR-yitM* Δ*sigW* strain was grown in 2×SG supplemented with or without 1 mM IPTG. (C) Comparison of the genetic organization in NCIB3610 and BEST195. Homologous genes are shown by boxes of the same color. Genes only present in NCIB3610 are shown in red bold. The deleted regions in the Δ*yitR-yitM* and Δ*nprB-yitM* mutants are shown below the gene map of NCIB3610.

To determine whether *yitPOM* encodes a toxin, we examined the effect of *yitPOM* overexpression on growth. We constructed the strain P_*spac*-hy_-*yitPOM*, which ectopically expresses *yitPOM* from the strong isopropyl β-D-thiogalactopyranoside (IPTG)-inducible, LacI-repressible *spac*-hy promoter [49] in the *amyE* locus on the chromosome. The wild-type and P_*spac*-hy_-*yitPOM* strains were grown with vigorous shaking in 2× Schaeffer’s sporulation medium plus glucose (2×SG) [50] supplemented with 1 mM IPTG, and the optical density at 600 nm (OD_600_) was measured over time. These strains showed no difference in growth from exponential phase to stationary phase (Fig 2A). Toxin-producing bacteria normally express cognate anti-toxin proteins against their toxins, and the effects of the toxins do not appear unless the anti-toxin genes are deleted [2, 37, 38, 40]. Since toxin and anti-toxin genes are simultaneously inserted into the genomes as exogenous genes and are frequently located close to each other in the genome [2, 37, 38, 40], genome comparison is a powerful tool to identify toxin/anti-toxin gene sets. To identify candidates for an anti-toxin gene against the putative toxin encoded by *yitPOM*, we compared the genetic organization of the 3610 and *B*. *subtilis* var. *natto* BEST195 strains, the latter of which lacks *yitPOM*. This comparison revealed that *yitPOM* appears to be inserted between *yitR* and *yitL* in the 3610 genome, along with *yizB* and *yitQ*, which are predicted to encode a transcriptional regulator and a membrane protein, respectively (Fig 2C). Since the SdpI anti-toxin protein is a membrane protein, YitQ was a candidate for an anti-toxin protein to the putative toxin, although YitQ has no similarity to SdpI. Based on the DNA sequence, *yizB* and *yitQ* are predicted to form an operon with a downstream gene, *yitR*, which also encodes a membrane protein. Althogh *yitR* is present in BEST195, we kept it as a second candidate for the anti-toxin protein. To test whether these genes encoded anti-YIT toxin, we attempted to disrupt these genes. However, we were concerned that deleting these candidate anti-toxin genes might cause severe growth defects by releasing the activity of the toxin encoded by the genomic *yitPOM* operon. Therefore, we constructed a Δ*yitR-yitM* deletion strain that lacks the entire region from *yitR* to *yitM*, which contains the candidate anti-toxin genes *yitR* and *yitQ*, the unknown repressor gene *yizB*, and the putative toxin-encoding *yitPOM* operon (Fig 2C). Furthermore, since the expression of the secondary resistance mechanism against the SDP toxin is induced by the alternative sigma factor SigW (σ^W^) [51], we also constructed a *sigW* deletion strain. Subsequently, either the Δ*yitR-yitM* and Δ*sigW* mutation alone or both mutations together were introduced into the P_*spac*-hy_-*yitPOM* strain. We compared the growth of these strains in 2×SG medium supplemented with 1 mM IPTG. While the P_*spac*-hy_-*yitPOM* Δ*yitR-yitM* and P_*spac*-hy_-*yitPOM* Δ*sigW* mutants grew normally, the P_*spac*-hy_-*yitPOM* Δ*yitR-yitM* Δ*sigW* mutant showed mild cell lysis 3 h after the end of exponential phase (Fig 2A). We confirmed that *yitPOM* expression caused this cell lysis, as cell lysis was only observed when *yitPOM* expression was induced with IPTG (Fig 2B). These results indicate that *yitPOM* expression leads to the production of a toxin that causes cell lysis in a mutant strain lacking the putative anti-toxin genes and *sigW*. The σ^W^-regulated genes include multiple antibiotic resistant genes, such as a peptide exporter, an SdpI homolog, fosfomycin resistance proteins, sublancin resistance proteins, and a penicillin-binding protein [51–54]. Some of these genes might contribute to resistance to the putative toxin produced by *yitPOM*.

**Fig 3:**
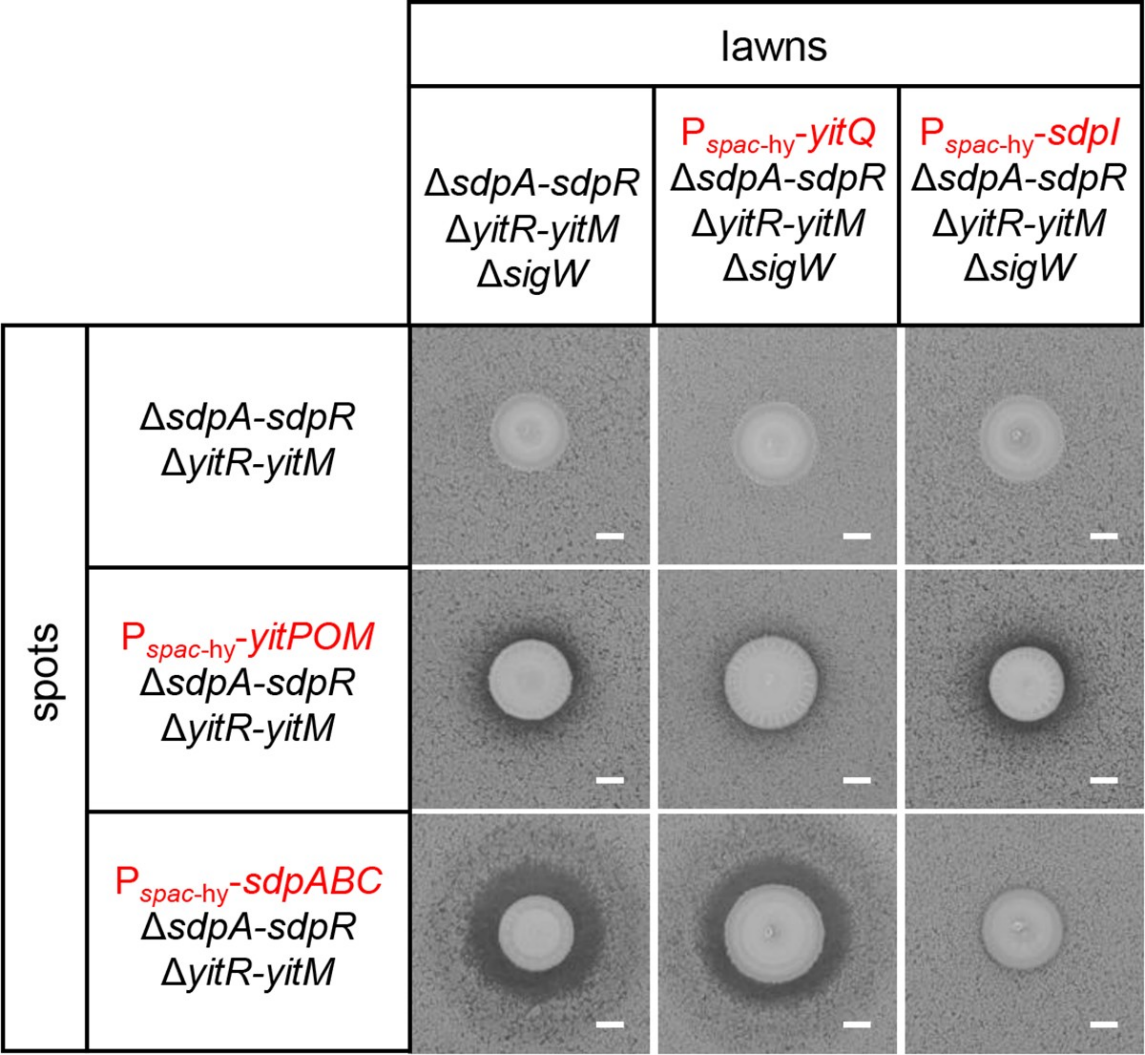
YitQ is an anti-toxin protein to the YIT toxin. *B*. *subtilis* strains Δ*sdpA-sdpR* Δ*yitR-yitM* Δ*sigW*, P_*spac*-hy_-*yitQ* Δ*sdpA-sdpR* Δ*yitR-yitM* Δ*sigW*, and P_*spac*-hy_-*sdpI* Δ*sdpA-sdpR* Δ*yitR-yitM* Δ*sigW* were added to 2×SG agar containing 1 mM IPTG and poured into plates as lawns. Strains tested for antibiotic production (shown on the left of the figure as spots) were spotted on the lawns. Plates were incubated at 37°C. A growth inhibitory zone was observed if the lawn strain was sensitive to a compound produced by the strain spotted on it. Scale bar, 2 mm.

To further confirm that *yitPOM* encodes a toxin, we employed a spot-on-lawn assay. We performed this assay in the Δ*sdpABC-sdpIR* (hereafter referred to as Δ*sdpA-sdpR*) Δ*yitR-yitM* mutant background to eliminate the effects of the endogenous *sdpABC* and *yitPOM* operons. Since the Δ*sigW* mutant is sensitive to multiple antibiotics and stresses, including the SDP toxin [51] and the putative toxin produced by *yitPOM*, we used the Δ*sdpA-sdpR* Δ*yitR-yitM* Δ*sigW* mutant as an antibiotic-sensitive indicator strain. When spotted on a lawn of this indicator strain, the strain expressing YitPOM (P_*spac*-hy_-*yitPOM* Δ*sdpA-sdpR* Δ*yitR-yitM*) formed growth inhibition zones (halos) around its colonies (Fig 3). By contrast, a strain that does not express YitPOM (Δ*sdpA-sdpR* Δ*yitR-yitM*) formed no obvious halos around its colonies on the same lawn. These results demonstrate that, like *sdpABC*, *yitPOM* encodes a secreted toxin, which we named YIT.

### YitQ is an anti-toxin protein to the YIT toxin

To determine whether YitQ is an anti-toxin protein to the YIT toxin, we examined the effect of *yitQ* overexpression on the YIT toxin activity. To this end, the P_*spac*-hy_-*yitQ* construct was introduced into the *amyE* locus of the indicator strain (i.e., the Δ*sdpA-sdpR* Δ*yitR-yitM* Δ*sigW* mutant). When spotted on a lawn of the indicator strain expressing YitQ (P_*spac*-hy_-*yitQ* Δ*sdpA-sdpR* Δ*yitR-yitM* Δ*sigW*), the strain expressing YitPOM (P_*spac*-hy_-*yitPOM* Δ*sdpA-sdpR* Δ*yitR-yitM*) only formed weak halos around its colonies (Fig 3). Thus, *yitQ* expression confered resistance to the YIT toxin.

We were interested in whether there is crosstalk between *yitPOM*/*yitQ* and *sdpABC*/*sdpI*. To explore this possibility, a strain expressing SdpABC (P_*spac*-hy_-*sdpABC* Δ*sdpA-sdpR* Δ*yitR-yitM*) was spotted onto lawns of the control indicator strain (Δ*sdpA-sdpR* Δ*yitR-yitM* Δ*spo0A*) and the indicator strain expressing YitQ (P_*spac*-hy_-*yitQ* Δ*sdpA-sdpR* Δ*yitR-yitM* Δ*sigW*) (Fig 3). The SdpABC-expressing strain formed clear halos around its colonies on both types of lawns. Thus, *yitQ* expression did not confer resistance to the SDP toxin. We also tested whether SdpI expression confers resistance to the YIT and SDP toxins. A strain expressing YitPOM (P_*spac*-hy_-*yitPOM* Δ*sdpA-sdpR* Δ*yitR-yitM*) formed halos around its colonies on a lawn of the indicator strain expressing SdpI (P_*spac*-hy_-*sdpI* Δ*sdpA-sdpR* Δ*yitR-yitM* Δs*igW*), whereas the strain expressing SdpABC (P_*spac*-hy_-*sdpABC* Δ*sdpA-sdpR* Δ*yitR-yitM*) did not. These results indicate that YitQ and SdpI are anti-toxin proteins specific to the YIT and SDP toxins, respectively. Thus, the two toxin/anti-toxin gene pairs *yitPOM*/*yitQ* and *sdpABC*/*sdpI* most likely function independently.

### Expression of *yitPOM* and *yitQ*

**Fig 4:**
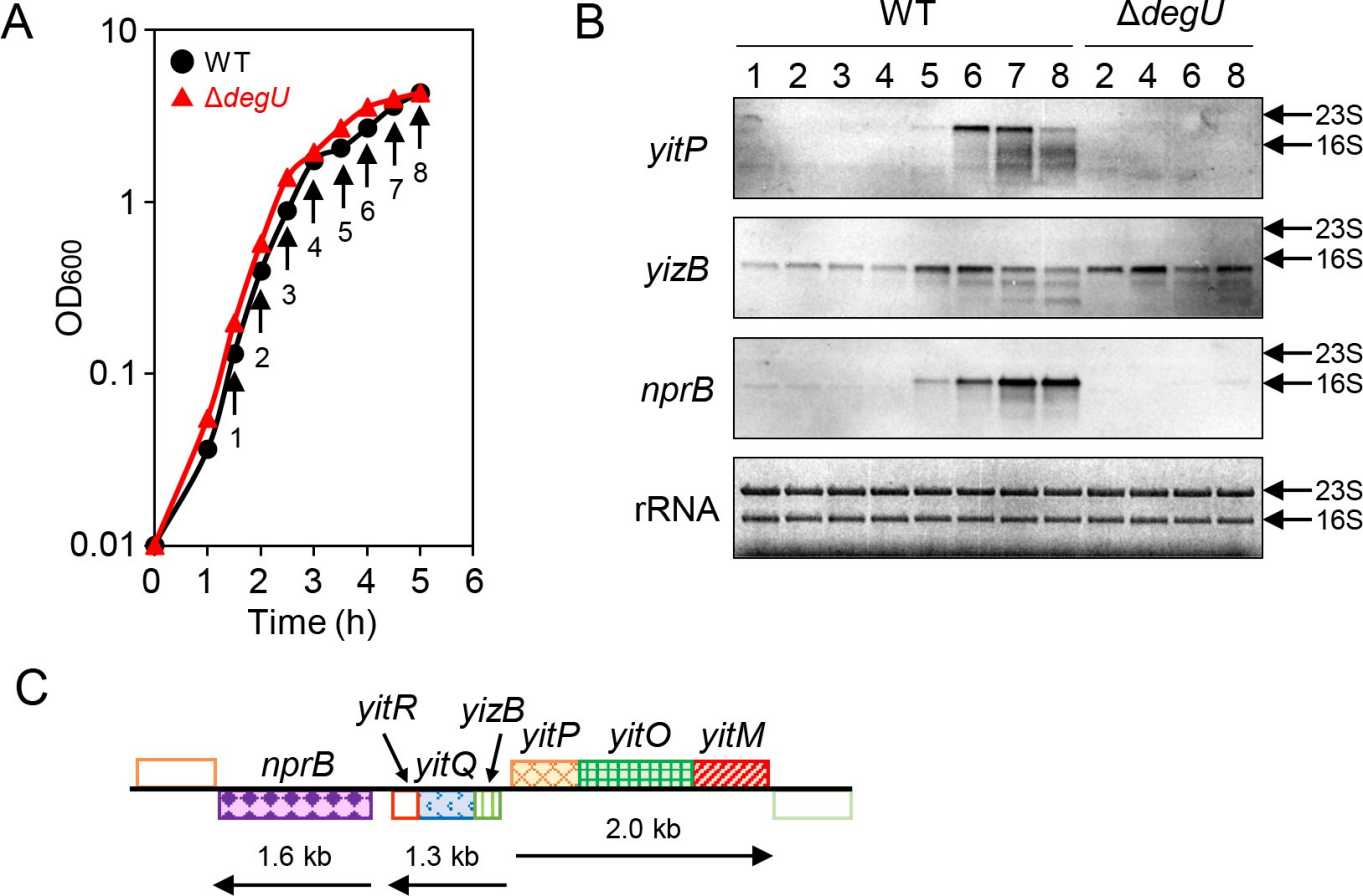
Transcription of *yitPOM* and *yitQ* in the Δ*degU* mutant. (A) Growth profiles of the wild-type and Δ*degU* mutant strains. Strains were grown in 2×SG with vigorous shaking. Arrows indicate the time points at which samples were taken for RNA isolation. (B) Northern blot analysis of *yitPOM*, *yizB-yitQ-yitR*, and *nprB*. Transcripts were detected with gene-specific DIG-labeled RNA probes. Lane numbers (time points) under the strain names correspond to the time points shown in panel A. rRNA stained with methylene blue is shown as a loading control. The positions of 23S rRNA and 16S rRNA are indicated by arrows. (C) The transcription map of the *yitPOM* region. The transcripts are represented as lines with arrows below the gene map, and their estimated lengths are indicated.

The *yitPOM* operon was previously identified as a member of the DegS-DegU-regulated genes via a DNA microarray analysis using another *B*. *subtilis* strain, ATCC6051 [47]. To confirm this property in strain 3610, we carried out a Northern blot analysis. RNA samples were isolated from wild-type and Δ*degU* mutant cells grown for various lengths of time in 2×SG with vigorous shaking (Fig 4A). We detected a single band at a position between the 23S rRNA (2904 nt) and 16S rRNA (1541 nt) on Northern blots with a *yitP*-specific probe (Fig 4B). The size of the band was consistent with the length of the entire *yitPOM* locus (2031 bp), confirming that the *yitPOM* locus is transcribed as an operon (Fig 4C). On the Northern blots, the *yitPOM* transcript was observed in the stationary phase samples from the wild-type strain but not in those from the Δ*degU* mutant (Fig 4B). These results indicate that DegS-DegU directly or indirectly induces *yitPOM* transcription in stationary phase.

*yitQ* is predicted to form an operon with its upstream and downstream genes, *yizB* and *yitR*. We detected a band below the position of 16S rRNA on Northern blots with a *yizB*-specific probe (Fig 3B). The size of the band was consistent with the length of the *yizB-yitQ*-*yitR* locus (1244 bp), supporting the conclusion that *yizB*, *yitQ*, and *yitR* are transcribed as an operon (Fig 4C). Based on the Northern blots, the *yizB-yitQ-yitR* operon was transcribed at low levels during exponential phase and then induced during stationary phase in the wild-type strain (Fig 4B). The Δ*degU* mutation had no significant effect on the transcription of the *yizB-yitQ-yitR* operon. The SDP toxin mediates cannibalism between “Spo0A ON” and “Spo0A OFF” cells [40]. The finding that DegS-DegU regulates *yitPOM* but not *yitQ* rules out the possibility that the YIT toxin mediates cannibalism between “DegU ON” and “DegU OFF” cells.

**Fig 5:**
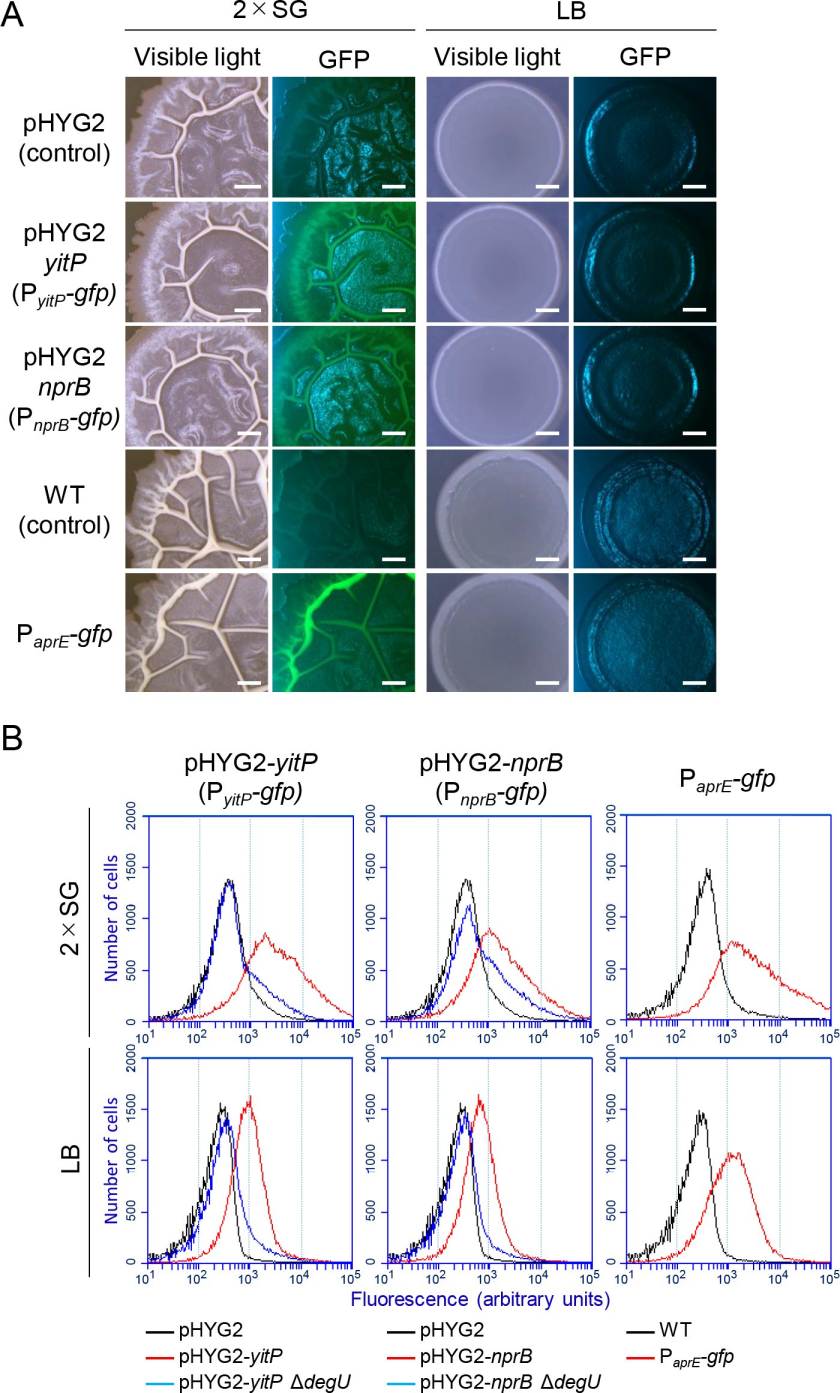
*yitPOM* expression is induced in biofilms by DegS-DegU. (A) Expression of *yitPOM* and *nprB* in biofilms. The wild-type strain 3610 carrying the multi-copy plasmid pHYG2 (promoterless *gfp*), pHYG2-*yitP* containing the P_*yitP*_-*gfp* reporter, or the single copy of *aprE-gfp* was grown at 37°C for 24 h on 2×SG or LB. GFP fluorescence was analyzed with a digital color camera. GFP fluorescence was observed as green light signals. Some excitation light reflections on the surfaces of colonies and media were observed as blue light signals on GFP images. Strains 3610 pHYG2 (promoter-less *gfp*) and 3610 were used as negative controls. Scale bar, 1 mm. (B) Flow cytometry analysis of *gfp* reporter strains. *B*. *subtilis* strains were grown at 37°C for 24 h on 2×SG or LB. Expression of *gfp* reporters in the colonies was analyzed using stains 3610 pHYG2 and 3610 as negative controls.

To explore the function of the YIT toxin, we asked under what conditions *yitPOM* expression is induced. The DegS-DegU-regulated gene *bpr*, which encodes an extracellular protease, is expressed in biofilms [55]. We therefore speculated that *yitPOM* is also expressed in biofilms. To visualize *yitPOM* expression in biofilms, the *yitP* promoter was fused to the green fluorescent protein (GFP) reporter, and the resulting P_*yitP*_*-gfp* reporter construct was introduced to the *amyE* locus on the chromosome of the wild-type strain. *B*. *subtilis* biofilms are wrinkled structures on the surfaces of colonies grown on solid media that support biofilm formation, such as 2×SG [24]; therefore, we attempted to examine the expression level of the P_*yitP*_*-gfp* reporter in colonies grown on 2×SG solid medium. However, we did not detect any fluorescent GFP signal on these colonies, probably because the *yitP* promoter activity was too low to detect signals in our microscopy. We next examined the expression of the P_*yitP*_*-gfp* reporter inserted into the multi-copy plasmid pHYG2. The expression of the P_*yitP*_*-gfp* reporter on the plasmid was examined at 37°C because the plasmid pHYG2-*yitP* negatively affected biofilm formation at 30°C. The wild-type strain carrying the multi-copy plasmid pHYG2-*yitP* (P_*yitP*_*-gfp*) formed wrinkled structures on the surfaces of colonies grown on 2×SG solid medium as the biofilms developed. Weak green GFP signal was observed in these wrinkles with a color digital camera (Fig 5A). By contrast, this strain formed flat colonies on LB medium, which does not support biofilm formation, and produced no detectable green GFP signal. Under the same conditions, the wild-type strain carrying the parental plasmid pHYG2 (promoterless *gfp*) produced no green fluorescent signal on either 2×SG or LB.

The expression of P_*yitP*_*-gfp* in these colonies was also analyzed at the single-cell level. Flow cytometry analysis revealed that the expression of P_*yitP*_*-gfp* was heterogeneous in the population in colonies grown on 2×SG and that the considerable portion of P_*yitP*_*-gfp* cells exhibited stronger fluorescence in 2×SG than in LB (Fig 5B). Combined with the microscopic observation, these results indicate that biofilms contains cells that highly express the *yitPOM* operon. Moreover, we confirmed that the expression of P_*yitP*_*-gfp* in these colonies was DegU-dependent as the level of fluorescence decreased to the background level in the Δ*degU* mutant (Fig 5B).

To eliminate potential artifacts resulting from multi-copy plasmid-based experiments, we examined the expression of *aprE*, which is one of the most highly expressed genes among the DegS-DegU-regulated genes [47]. We used a strain carrying a single copy of the P_*aprE*_*-gfp* reporter inserted into the *amyE* locus on the chromosome. The P_*aprE*_*-gfp* strain produced GFP fluorescent signal in the wrinkles of colonies grown on 2×SG (Fig 5A). By contrast, no detectable GFP signal was observed when the P_*aprE*_*-gfp* strain was grown on LB. Flow cytometry analysis revealed that the considerable portion of P_*aprE*_*-gfp* cells exhibited stronger fluorescence in 2×SG than in LB (Fig 5B). Thus, the expression profiles of P_*aprE*_*-gfp* were quite similar to those of P_*yitP*_*-gfp*. These results indicate that DegS-DegU strongly induces its regulatory target genes, including *yitPOM*, in biofilms and that the YIT toxin may play a role in biofilms.

### The YIT toxin inhibits colony biofilm formation

**Fig 6:**
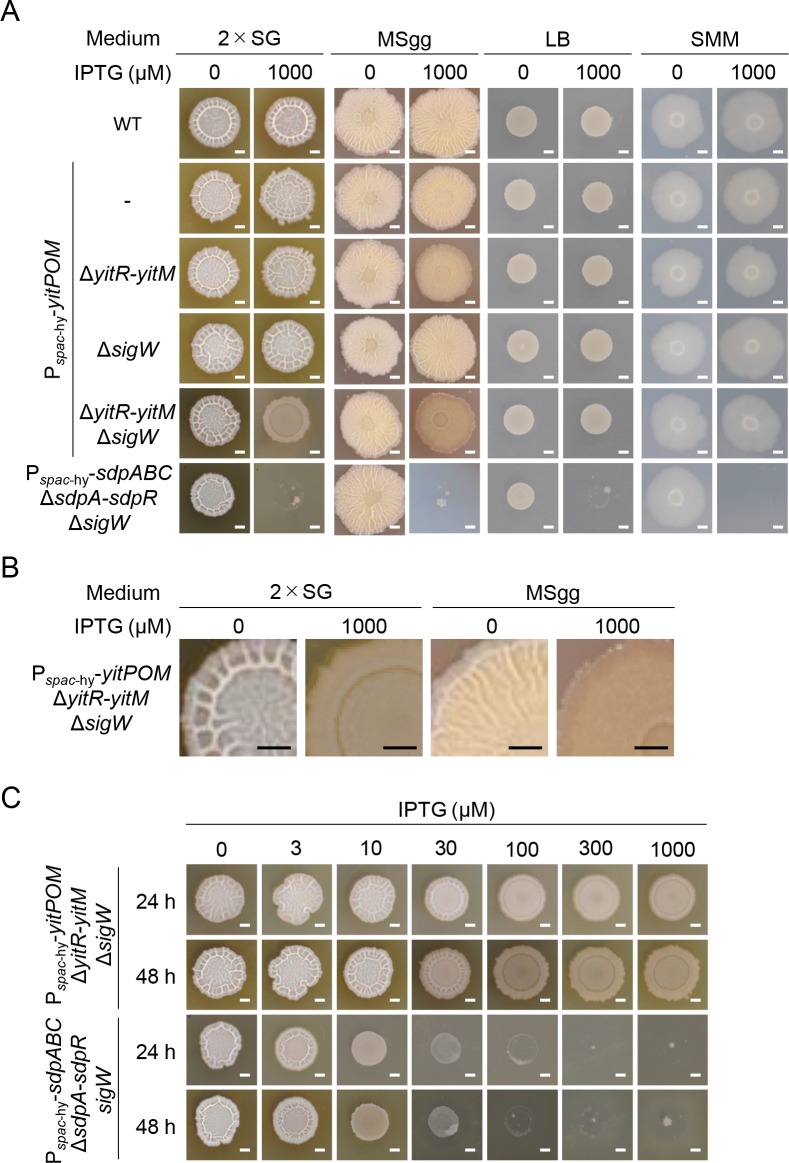
Expression of *yitPOM* inhibits biofilm formation. (A) P_*spac*-hy_-*yitPOM* strains with the indicated mutations were grown at 30°C for 48 h on biofilm formation media (2×SG and MSgg) and non-biofilm formation media (LB and SMM) with or without 1000 μM IPTG. Colonies of the wild-type and P_*spac*-hy_-*sdpABC* Δ*sdpA-sdpR* Δ*sigW* strains are also shown as references. (B) Magnified images of the P_*spac*-hy_-*yitPOM* Δ*yitR-yitM* Δ*sigW* mutant colonies shown in panel A. (C) Comparison of the effects of *yitPOM* and *sdpABC* overexpression on colony morphology. The P_*spac*-hy_-*yitPOM* Δ*yitR-yitM* Δ*sigW* and P_*spac*-hy_-*sdpABC* Δ*sdpA-sdpR* Δ*sigW* mutant strains were grown at 30°C for 48 h on 2×SG with various IPTG concentrations. Colony morphology analysis was done at least three time, and the typical examples were shown in the figure. Scale bar, 2 mm.

We hypothesized that if the YIT toxin has an effect on biofilm formation, we expected to detect this effect on biofilm supporting media but not on the media that do not support biofilm formation. First, we examined whether *yitPOM* overexpression from the *spac*-hy promoter affects colony biofilm formation on biofilm-supporting media. We used two biofilm-supporting media, the rich complex medium 2×SG and the synthetic medium MSgg [24]. On 2×SG solid medium, the wild-type strain formed whitish wrinkled colonies (Fig 6A). Induction of *yitPOM* did not affect the colony morphologies of the wild-type, Δ*yitR-yitM* mutant, or Δ*sigW* mutant strains; these P_*spac*-hy_-*yitPOM* strains formed similar whitish wrinkled colonies in the presence or absence of IPTG. By contrast, *yitPOM* induction altered the colony morphology of the Δ*yitR-yitM* Δ*sigW* mutant; the P_*spac*-hy_-*yitPOM* Δ*yitR-yitM* Δ*sigW* mutant formed brown flat colonies in the presence of IPTG (Fig 6A). Magnified images showed that the whitish wrinkled layers (biofilms) were completely absent on the surfaces of the P_*spac*-hy_-*yitPOM* Δ*yitR-yitM* Δ*sigW* mutant colonies in the presence of IPTG (Fig 6B). Similar results were obtained on MSgg medium. The wild-type strain formed light brown wrinkled colonies on MSgg (Fig 6A). Induction of *yitPOM* altered the colony morphology of the Δ*yitR-yitM* Δ*sigW* mutant. The P_*spac*-hy_-*yitPOM* Δ*yitR-yitM* Δ*sigW* mutant formed brown flat colonies in the presence of IPTG (Fig 6A), and these colonies completely lacked the light brown wrinkled layers (biofilms) on their surfaces (Fig 6B). Unlike on rich 2×SG medium, induction of *yitPOM* also altered the colony morphology of the Δ*yitR-yitM* mutant when it was grown on MSgg (Fig 6A). In the presence of IPTG, the P_*spac*-hy_-*yitPOM* Δ*yitR-yitM* mutant formed colonies covered with attenuated wrinkles at 96 h post-inoculation; however, these wrinkles faded over time (S1 Fig). This phenotype suggests that YitQ may play a major role in resistance to the YIT toxin under low nutrient conditions, such as *B*. *subtilis* natural habitats, soils. We next examined the colony morphologies on the complex medium LB and on the synthetic medium Spizizen minimal medium (SMM) [56]. *B*. *subtilis* forms flat colonies rather than biofilms on these media. On these media, *yitPOM* induction had little or no effect on colony morphology, even in the Δ*yitR-yitM* Δ*sigW* mutant (Fig 6A). We compared the effect of *yitPOM* overexpression on colony morphology with that of *sdpABC* overexpression. For this, *sdpABC* was expressed under the control of the same promoter (*spac*-hy) in the Δ*sdpA-sdpR* Δ*sigW* mutant. The P_*spac*-hy_-*sdpABC* Δ*sdpA-sdpR* Δ*sigW* mutant formed normal colonies in all four media in the absence of IPTG, but it did not form colonies in the presence of IPTG (Fig 6A). Thus, the SDP toxin inhibited overall cell growth independently of the medium conditions.

We investigated the relationship between the expression levels of *yitPOM* and *sdpABC* and colony morphology. For this purpose, P_*spac*-hy_-*yitPOM* Δ*yitR-yitM* Δ*sigW* and P_*spac*-hy_-*sdpABC* Δ*sdpA-sdpR* Δ*sigW* mutants were grown on 2×SG medium supplemented with various IPTG concentrations (0 to 1000 μM) (Fig 6C). The effect of *yitPOM* expression on colony morphology appeared when the P_*spac*-hy_-*yitPOM* Δ*yitR-yitM* Δ*sigW* mutant was grown in the presence of 30 μM or higher IPTG concentrations. At 30 μM IPTG, attenuated wrinkles appeared on the colonies at 24 h post-inoculation; however, these wrinkles failed to grow further. At 100 μM IPTG, the colonies completely lacked the whitish wrinkled layers on their surface and became flat. Higher IPTG concentrations did not further alter the colony morphology. Despite having an obvious effect on colony morphology, *yitPOM* induction did not affect colony size. By contrast, when grown on 2×SG medium supplemented with various IPTG concentrations, the P_*spac*-hy_-*sdpABC* Δ*sdpA-sdpR* Δ*sigW* mutant formed small colonies in the presence of 10 or 30 μM IPTG but did not form colonies at 100 μM or higher IPTG concentrations (Fig 6C). Thus, *sdpABC* expression exerted a stronger effect on colony formation as its expression levels increased. These results demonstrate that the YIT and SDP toxins have different effects on colony growth and that the YIT toxin specifically inhibits biofilm formation in the absence of its resistance genes.

We considered how the YIT toxin inhibits biofilm formation. As described above, *yitPOM* induction caused mild cell lysis only in Δ*yitR-yitM* Δ*sigW* mutant cells grown in 2×SG medium with shaking. Induction of *yitPOM* in the Δ*yitR-yitM* Δ*sigW* mutant did not cause cell lysis in cultures grown in LB medium with shaking (S2 Fig). Thus, *yitPOM* induction caused cell lysis and inhibition of biofilm formation only in the Δ*yitR-yitM* Δ*sigW* mutant cells grown on biofilm formation media, indicating that these two phenotypes represent different aspects of one phenomenon. Moreover, induction of *yitPOM* led to formation of halos in the spot-on-lawn assays. We propose that the YIT toxin likely inhibits biofilm formation by killing biofilm-forming cells rather than by preventing expression of biofilm formation genes.

### NprB allows the YIT toxin to attack cells within biofilms

**Fig 7:**
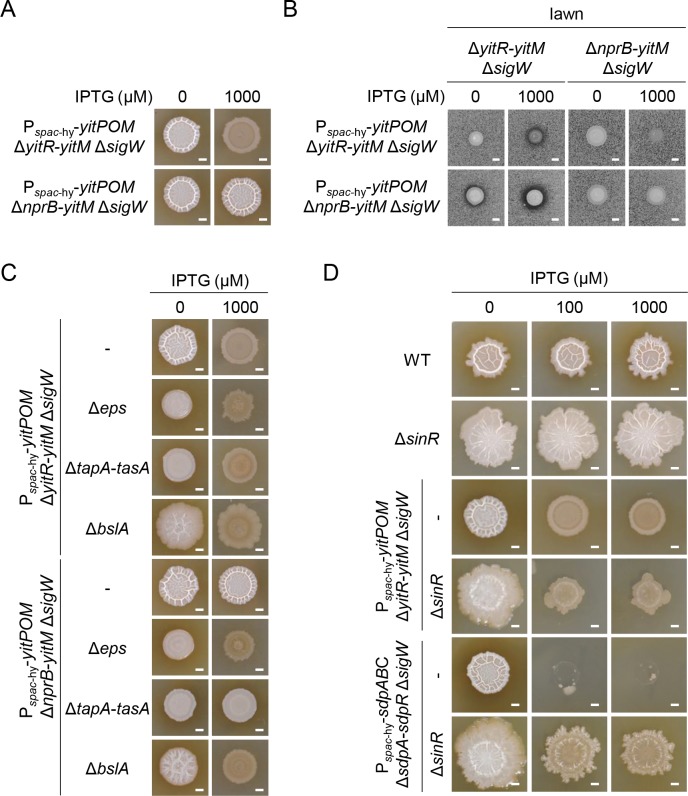
The NprB protease is required for the YIT toxin to inhibit biofilm formation. (A) The Δ*nprB* mutation prevents the YIT toxin from inhibiting biofilm formation. P_*spac*-hy_-*yitPOM* Δ*yitR-yitM* Δ*sigW* and P_*spac*-hy_-*yitPOM* Δ*nprB-yitM* Δ*sigW* cells were grown on 2×SG at 30°C for 48 h with or without 1000 μM IPTG. (B) Production of the YIT toxin. Δ*yitR-yitM* Δ*sigW* and Δ*nprB-yitM* Δ*sigW* cells were added to 2×SG 1.2% agar with or without 1000 μM IPTG, and the mixtures were poured into plates. P_*spac*-hy_-*yitPOM* Δ*yitR-yitM* Δ*sigW* and P_*spac*-hy_-*yitPOM* Δ*nprB-yitM* Δ*sigW* cells were spotted on these lawn plates. The plates were then incubated at 37°C for 24 h. (C) The Δ*eps* and Δ*bslA* mutations bypass the requirement for NprB in the ability of the YIT toxin to inhibit biofilm formation. (D) Overproduction of biofilm matrix polymers interfered with the action of the SDP toxin but not with that of the YIT toxin. Scale bar, 2 mm.

Induction of *yitPOM* exerted its effects only in cells grown on biofilm formation media. However, the *spac*-hy promoter is active in rich and poor media, including LB and SMM, as observed for the P_*spac*-hy_-*sdpABC* Δ*sdpA-sdpR* Δ*sigW* mutant (Fig 6A). These observations suggest the involvement of other factor(s) in the functions of the YIT toxin. A comparison of the genetic organization of the 3610 and BEST195 strains revealed that, in addition to *yizB* and *yitQ*, *nprB* appears to be inserted into the 3610 genome along with *yitPOM* (Fig 2C). Like *yitPOM*, *nprB*, which encodes an extracellular neutral protease, was transcribed in a DegU-dependent manner (Fig 4B), and its expression was induced in biofilms (Fig 5A and 5B). To determine whether *nprB* is involved in the YIT toxin function, we introduced a deletion of the *nprB-yitM* region (Fig 2C) into the P_*spac*-hy_-*yitPOM* Δ*sigW* mutant and examined the colony morphology of the resulting strain. Unlike in the P_*spac*-hy_-*yitPOM* Δ*yitR-yitM* Δ*sigW* mutant, *yitPOM* induction did not inhibit biofilm formation in the P_*spac*-hy_-*yitPOM* Δ*nprB-yitM* Δ*sigW* mutant. This strain formed whitish wrinkled colonies like those of the wild-type strain in the presence or absence of IPTG (Fig 7A). Because *nprB* deletion was the only genetic difference between P_*spac*-hy_-*yitPOM* Δ*yitR-yitM* Δ*sigW* and P_*spac*-hy_-*yitPOM* Δ*nprB-yitM* Δ*sigW* mutants (Fig 2C), this result suggests that the NprB protease is required for the production or function of the YIT toxin.

To distinguish these possibilities, we examined the production of the YIT toxin in these mutants via spot-on-lawn assays. The P_*spac*-hy_-*yitPOM* Δ*yitR-yitM* Δ*sigW* and P_*spac*-hy_-*yitPOM* Δ*nprB-yitM* Δ*sigW* mutants were spotted on the lawn of the Δ*yitR-yitM* Δ*sigW* mutant. Although both mutants formed halos around their colonies in the presence of IPTG, the P_*spac*-hy_-*yitPOM* Δ*nprB-yitM* Δ*sigW* mutant formed clearer halos than did the P_*spac*-hy_-*yitPOM* Δ*yitR-yitM* Δ*sigW* mutant (Fig 7B). The P_*spac*-hy_-*yitPOM* Δ*yitR-yitM* Δ*sigW* mutant formed smaller colonies on the lawn than did the P_*spac*-hy_-*yitPOM* Δ*nprB-yitM* Δ*sigW* mutant, likely due to loss of biofilm formation. Therefore, we compared the YIT toxin production between the P_*spac*-hy_-*yitPOM* Δ*yitR-yitM* and P_*spac*-hy_-*yitPOM* Δ*nprB-yitM* mutants. Although these mutants formed similar colonies on the lawn of the Δ*yitR-yitM* Δ*sigW* mutant, the P_*spac*-hy_-*yitPOM* Δ*nprB-yitM* mutant formed clearer halos than did the P_*spac*-hy_-*yitPOM* Δ*yitR-yitM* mutant (S3 Fig). Thus, the Δ*nprB* mutation increased YIT toxin production or activity. These results suggest that NprB is not required for YIT toxin production. Given that NprB is an extracellular neutral protease, these results suggest that the YIT toxin is probably a substrate for NprB.

We next examined the alternative possibility that NprB might be required for the function of the YIT toxin. To test this idea, we spotted the P_*spac*-hy_-*yitPOM* Δ*yitR-yitM* Δ*sigW* and P_*spac*-hy_-*yitPOM* Δ*nprB-yitM* Δ*sigW* mutants on the lawn of the Δ*nprB-yitM* Δ*sigW* mutant. Both mutants failed to form clear halos around their colonies (Fig 7B), supporting this idea.

We explored why the Δ*nprB* mutation impaired the function of the YIT toxin. Cells in biofilms are covered with and protected by biofilm matrix polymers, a key reason why cells in biofilms exhibit increased antibiotic tolerance or resistance [5, 6]. We hypothesized that a similar mechanism might work against the YIT toxin and that the NprB protease might enable the YIT toxin molecules to pass through the layers of the biofilm matrix polymers to attack cells within the biofilms. If this were true, then disrupting the biofilm matrix would enable the YIT toxin to inhibit biofilm formation even in the Δ*nprB* mutant. The biofilm matrix of *B*. *subtilis* biofilms mainly consists of exopolysaccharides (synthesized by the products of the *eps* operon) and polymers of the TasA (produced by the *tapA-tasA* operon) and BslA proteins [24–29]. To test our hypothesis, we introduced Δ*eps*, Δ*tapA-tasA*, and Δ*bslA* deletion mutations into the P_*spac*-hy_-*yitPOM* Δ*yitR-yitM* Δ*sigW* and P_*spac*-hy_-*yitPOM* Δ*nprB-yitM* Δ*sigW* mutants and examined their colony morphologies. The P_*spac*-hy_-*yitPOM* Δ*yitR-yitM* Δ*sigW* Δ*eps* mutant formed whitish mucoid colonies in the absence of IPTG, while it formed flat brown colonies in the presence of IPTG (Fig 7C). The difference in colony morphology depending on the presence or absence of IPTG indicates that the induced YIT toxin can function in these colonies even though the Δ*eps* mutation impaired biofilm formation and led to the formation of mucoid colonies. Unlike the P_*spac*-hy_-*yitPOM* Δ*nprB-yitM* Δ*sigW* mutant, the P_*spac*-hy_-*yitPOM* Δ*nprB-yitM* Δ*sigW* Δ*eps* mutant also formed whitish mucoid colonies in the absence of IPTG and flat brown colonies in the presence of IPTG (Fig 7C). Thus, the Δ*nprB* mutation did not interfere with the function of the YIT toxin in the Δ*eps* mutant. Similar results were obtained with the Δ*bslA* mutant. The P_*spac*-hy_-*yitPOM* Δ*yitR-yitM* Δ*sigW* Δ*bslA* and P_*spac*-hy_-*yitPOM* Δ*nprB-yitM* Δ*sigW* Δ*bslA* mutants formed whitish mucoid colonies in the absence of IPTG and flat brown colonies in the presence of IPTG (Fig 7C). These results demonstrate that the Δ*eps* and Δ*bslA* mutations eliminate the requirement for NprB in the function of the YIT toxin. Thus, our idea that NprB enables the YIT toxin to pass through the layers of the biofilm matrix polymers to attack cells in the biofilm is very likely. On the other hand, the Δ*tapA-tasA* mutation did not restore the YIT toxin activity in the Δ*nprB-yitM* mutant. The P_*spac*-hy_-*yitPOM* Δ*nprB-yitM* Δ*sigW* Δ*tapA-tasA* mutants formed whitish mucoid colonies in the presence or absence of IPTG (Fig 7C). These results indicate that exopolysacchrides, BslA polymers or molecules associated with these polymers probably trap the YIT toxin in Δ*nprB* mutant biofilms.

We further examined the effect of overexpression of biofilm matrix polymers on the YIT toxin activity. SinR is a major repressor of the biofilm matrix synthesis genes [32], and a Δ*sinR* mutant formed large swollen colonies due to overproduction of biofilm matrix polymers (Fig 7D). We introduced the Δ*sinR* mutation into the P_*spac*-hy_-*yitPOM* Δ*yitR-yitM* Δ*sigW* mutant and examined the colony morphology of the resulting strain. In the absence of IPTG, the P_*spac*-hy_-*yitPOM* Δ*yitR-yitM* Δ*sigW* Δ*sinR* mutant formed large swollen colonies, like those of the Δ*sinR* mutant. Induction of *yitPOM* inhibited biofilm formation in this mutant. The P_*spac*-hy_-*yitPOM* Δ*yitR-yitM* Δ*sigW* Δ*sinR* mutant formed flat brown colonies in the presence of 100 or 1000 μM IPTG (Fig 7D), as was also observed in the P_*spac*-hy_-*yitPOM* Δ*yitR-yitM* Δ*sigW* mutant (Fig 6C). We also examined the effect of the Δ*sinR* mutation on the SDP toxin. The P_*spac*-hy_-*sdpABC* Δ*sdpA-R* Δ*sigW* Δ*sinR* mutant formed large swollen colonies in the absence of IPTG. Induction of *sdpABC* did not inhibit colony formation and only partly suppressed the swollen colony phenotype even in the presence of 1000 μM IPTG (Fig 7D). These results indicate that overproduction of biofilm matrix polymers interferes with the activity of the SDP toxin but not with that of the YIT toxin. Based on these results, we conclude that the YIT toxin can function within mature biofilms with the assistance of the extracellular neutral protease NprB.

**Fig 8:**
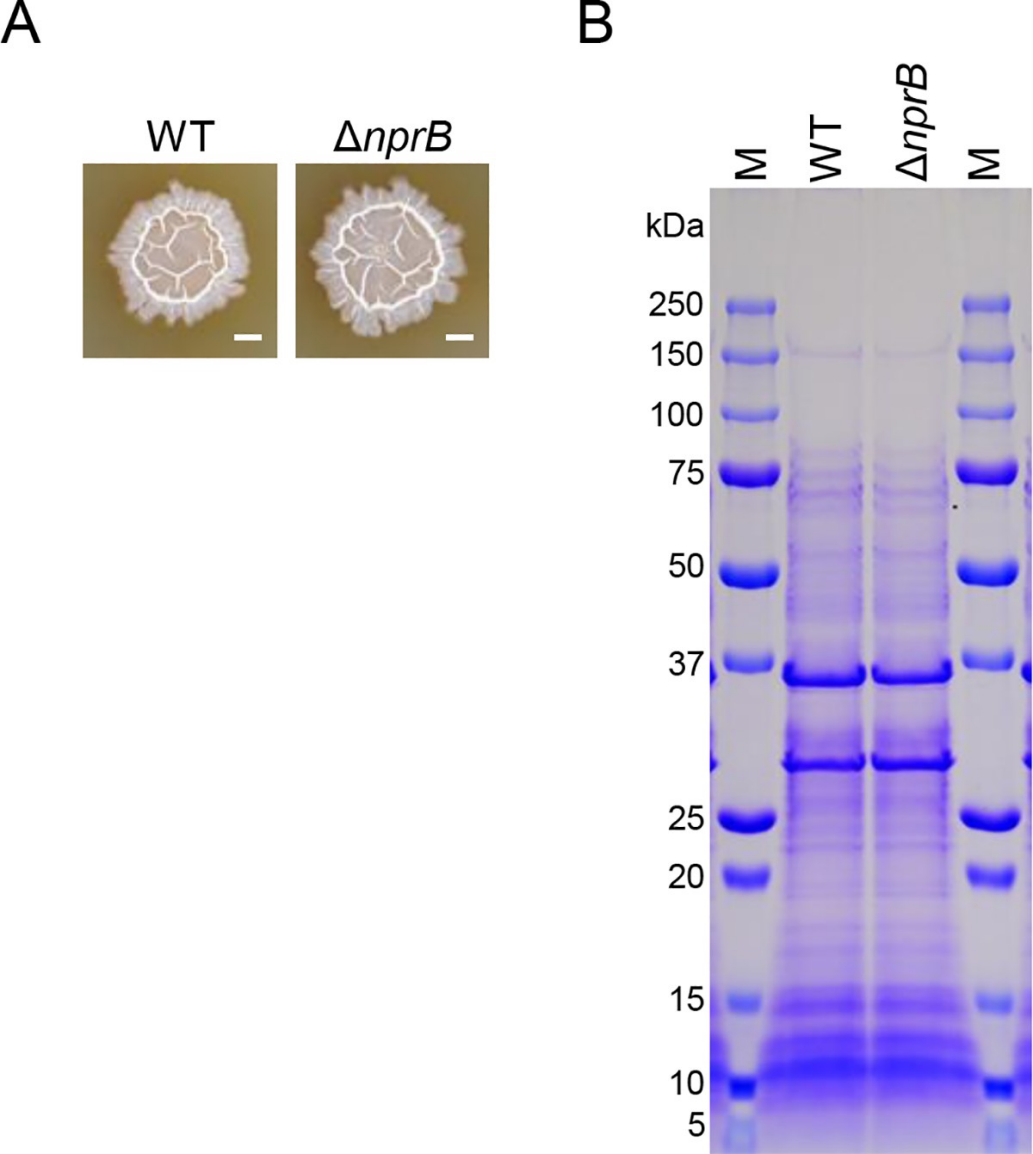
The Δ*nprB* mutation had no significant effect on biofilm formation. (A) Colony biofilms of the wild-type and Δ*nprB* mutant strains. These strains were grown at 30°C for 48 h on 2×SG. Scale bar, 2 mm. (B) The Δ*nprB* mutation had no significant effect on the composition of the extracellular proteins of colony biofilms. Colonies grown at 30°C for 48 h on 2×SG were suspended in SDS-PAGE sample buffer (62.5 mM Tris-HCl (pH 6.8), 1% SDS, 10% glycerol, 2.5% 2-mercaptoethanol, 2 mM PMSF, and 5 mM EDTA) and boiled for 2 min. After centrifugation, the supernatants were subjected to SDS-PAGE. Protein bands were visualized via Coomassie brilliant blue (CBB) staining. The size of protein molecular weight markers (lanes M) is indicated on the left side. The experiment was done twice, and the typical example was shown.

We examined the colony morphology of the Δ*nprB* mutant. The Δ*nprB* mutant formed wrinkled colonies on 2×SG medium similar to those of the wild-type strain (Fig 8A). We extracted the extracellular proteins and cell surface-associated proteins from these colony biofilms and analyzed them via SDS-PAGE. We detected little or no difference in the protein composition between the wild-type and Δ*nprB* mutant strains in the gels after Coomassie brilliant blue (CBB) staining (Fig 8B). These results indicate that, despite a clear effect on the function of the YIT toxin, the Δ*nprB* mutation does not significantly alter biofilm structure.

### The YIT toxin is present in biofilms of the wild-type strain

**Fig 9:**
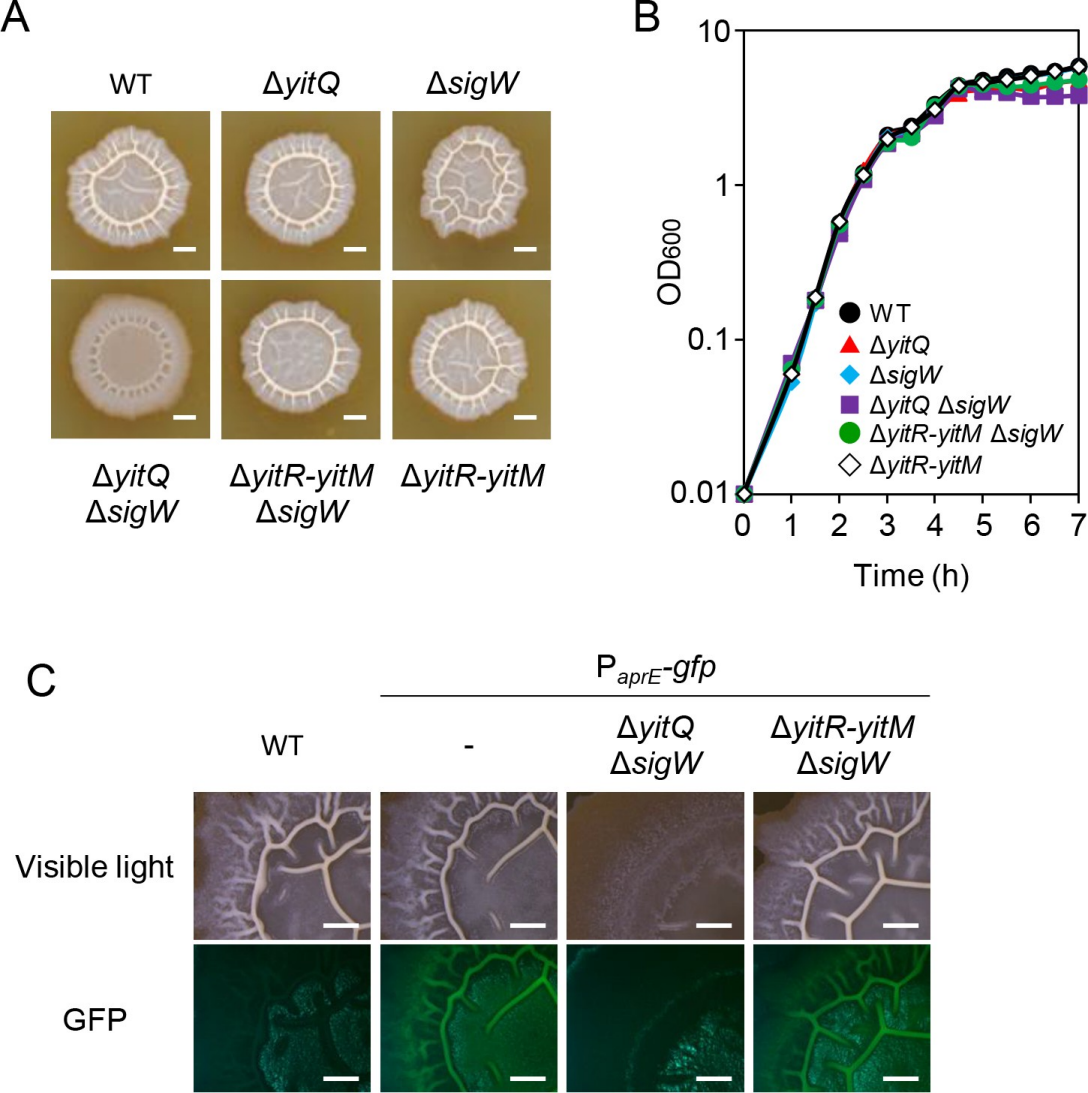
The YIT toxin is expressed and functions in the wild-type strain. (A) Colony morphologies of mutants lacking the resistance genes to the YIT toxin. The strains were grown at 30°C for 48 h on 2×SG. Scale bar, 2 mm. (B) Growth profiles in 2×SG shaking cultures. (C) P_*aprE*_-*gfp* expression was reduced in the Δ*yitQ* Δ*sigW* mutant. Scale bar, 1 mm.

So far, we have reported the results of experiments designed to uncover the function of the YIT toxin via *yitPOM* expression from the strong *spac*-hy promoter. We asked whether *yitPOM* expression from its own promoter is sufficiently high to exhibit the phenotypes observed above. As described above, the action of the YIT toxin was antagonized by YitQ and unidentified σ^W^-regulated gene product(s) in the wild-type strain. If the YIT toxin is present in biofilms of the wild-type strain, its effect should appear in *yitQ* and *sigW* mutants. Therefore, we examined the colony morphologies of mutants lacking *yitQ* and/or *sigW* on 2×SG medium (Fig 9A). While the Δ*yitQ* and Δ*sigW* single mutants formed whitish wrinkled colonies like those of the wild-type strain, the Δ*yitQ* Δ*sigW* double mutant formed colonies with attenuated wrinkles (biofilms). The Δ*yitR-yitM* Δ*sigW* mutant, which lacks both the toxin and anti-toxin genes, formed whitish wrinkled colonies like those of the wild-type strain. Thus, the phenotype of the Δ*yitQ* Δ*sigW* mutant was caused by the YIT toxin. However, the phenotype of the Δ*yitQ* Δ*sigW* mutant was slightly less noticeable than that of the P_*spac*-hy_-*yitPOM* Δ*yitR-yitM* Δ*sigW* mutant in the presence of IPTG. Likewise, when grown in shaking culture, the Δ*yitQ* Δ*sigW* mutant did not display the culture lysis phenotype as observed for the P_*spac*-hy_-*yitPOM* Δ*yitR-yitM* Δ*sigW* mutant; instead, the Δ*yitQ* Δ*sigW* mutant reached slightly lower OD_600_ in the stationary phase than that of the wild-type strain (Fig 9B).

As mentioned above, the P_*aprE*_*-gfp* reporter is induced in biofilms by DegS-DegU. The wild-type strain and the Δ*yitR-yitM* Δ*sigW* mutant with the P_*aprE*_*-gfp* reporter displayed bright GFP signal at wrinkles on colonies. However, the Δ*yitQ* Δ*sigW* mutant with the P_*aprE*_*-gfp* reporter displayed no detectable GFP signal on its colonies with attenuated wrinkles (Fig 9C). Thus, the Δ*yitQ* Δ*sigW* mutant showed decreased biofilm formation and decreased *aprE* expression. These results indicate that the YIT toxin reduces the number of biofilm-forming cells that express DegS-DegU-regulated genes, including *yitPOM*, in the Δ*yitQ* Δ*sigW* mutant. In other words, the action of the YIT toxin reduces the number of cells producing the YIT toxin in the Δ*yitQ* Δ*sigW* mutant. This effect can explain why the phenotype of the Δ*yitQ* Δ*sigW* mutant was slightly less obvious. These results demonstrate that the YIT toxin is present within biofilms of the wild-type strain.

### The YIT toxin can mediate intercellular competition within biofilms

We hypothesized that the YIT toxin might mediate intercellular competition within biofilms. To test this hypothesis, we designed the following experiment. Dilutions of cultures of the wild-type and Δ*yitR-yitM* Δ*sigW* mutant strains were mixed, and the mixtures were spotted on 2×SG solid medium. The inoculated cells grew and formed biofilms in which the wild-type cells were expected to produce the YIT toxin. The YIT toxin then exerted its effect in biofilms with the assistance of NprB, which expressed in wild-type and Δ*yitR-yitM* Δ*sigW* mutant biofilms. If the YIT toxin suppressed the growth of Δ*yitR-yitM* Δ*sigW* mutant cells within biofilms, the ratio of these strains within the biofilms would change from the initial ratio. To estimate the ratio of two strains within biofilms, the P_*aprE*_*-gfp* reporter was introduced into one strain to detect its cells within biofilms.

**Fig 10:**
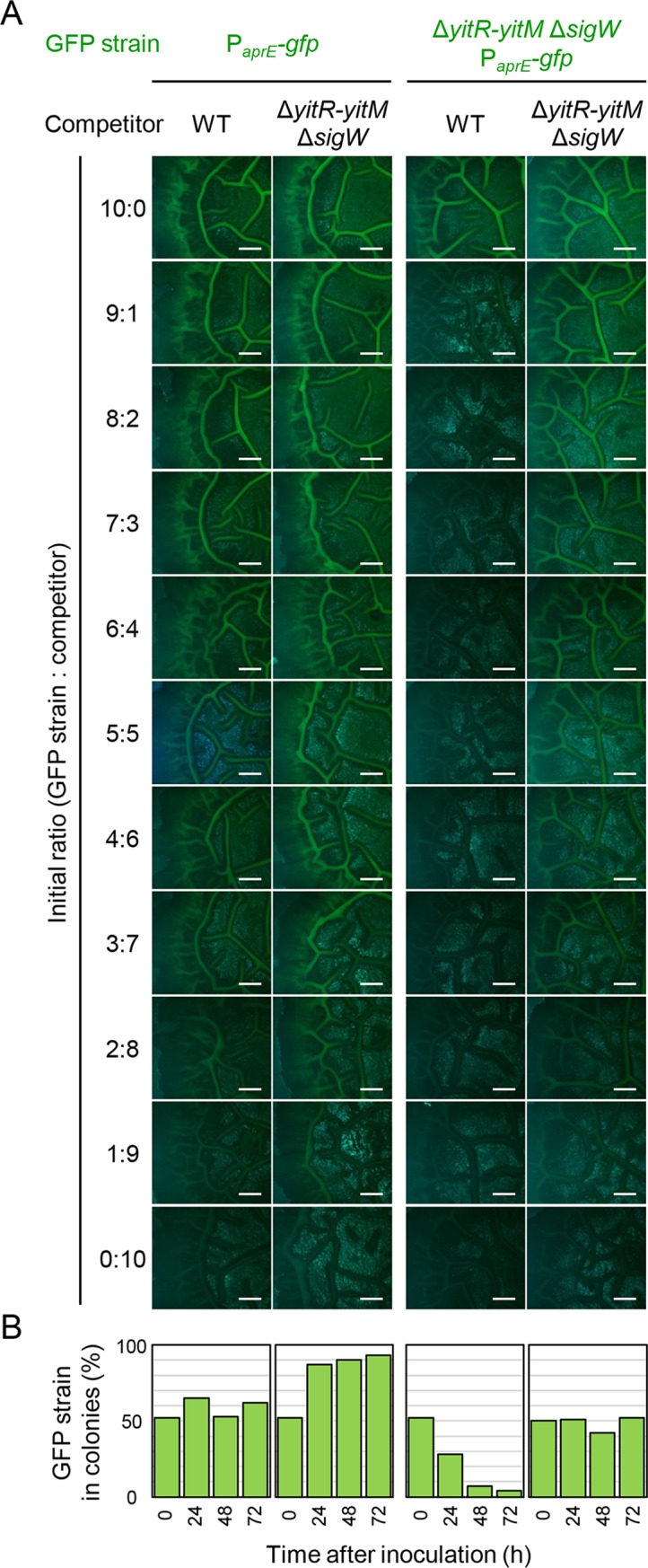
The YIT toxin mediates competition within biofilms. (A) P_*aprE*_-*gfp* expression in mixed colonies. Dilutions of cultures of the indicated strains were mixed at various ratios (10:0 to 0:10) and spotted on 2×SG. P_*aprE*_-*gfp* expression in the resultant colonies was analyzed 48 h after inoculation. The fluorescent images of colonies are shown. Scale bar, 1 mm. (B) The population ratios of P_*aprE*_-*gfp* cells in mixed colonies. Dilutions of cultures of the indicated strains on the top of the panel (A) were mixed at the ratio of 1:1, and spotted on 2×SG. The population ratios (%) of P_*aprE*_-*gfp* cells (Cm^r^) in the resultant colonies were analyzed over time by determining the numbers of Cm^r^ CFUs and total CFUs in colonies. The values are the average of 4 independent colony measurements.

First, wild-type cells carrying the P_*aprE*_*-gfp* reporter (the P_*aprE*_*-gfp* strain) were mixed with wild-type cells at various ratios from 10:0 to 0:10, and the mixtures were spotted on 2×SG solid medium. After 2 days of incubation, we observed the colonies with a fluorescence stereomicroscope. A bright GFP fluorescent signal was detected on colonies grown from the 10:0 mixture, and the GFP signal decreased as the proportion of the P_*aprE*_*-gfp* strain decreased (Fig 10A). When P_*aprE*_*-gfp*-expressing cells were mixed with Δ*yitR-yitM* Δ*sigW* mutant cells and grown on 2×SG medium, the GFP signal on the colonies also decreased as the proportion of the P_*aprE*_*-gfp* strain decreased; however, its decrease was moderate compared with that in the former experiment. For example, at a ratio of 3:7, obvious GFP signal was observed on colonies grown from the mixture of the *aprE-gfp* and Δ*yitR-yitM* Δ*sigW* mutant strains but not on colonies grown from the mixture of the *aprE-gfp* and wild-type strains. We also introduced the P_*aprE*_*-gfp* reporter into the Δ*yitR-yitM* Δ*sigW* mutant. When Δ*yitR-yitM* Δ*sigW* P_*aprE*_*-gfp* mutant cells were mixed with wild-type cells, no GFP signal was observed, even on the colonies grown from the 9:1 mixture (Fig 10A). By contrast, when Δ*yitR-yitM* Δ*sigW* P_*aprE*_*-gfp* mutant cells were mixed with Δ*yitR-yitM* Δ*sigW* mutant cells, the GFP signal on the colonies decreased as the proportion of the Δ*yitR-yitM* Δ*sigW* P_*aprE*_*-gfp* mutant cells decreased, as was also the case with the mixture of the *aprE-gfp* and wild-type strains. Thus, the YIT toxin is likely to mediate intercellular competition within biofilms.

To confirm these results, we analyzed the population of cells carrying the *aprE-gfp* reporter in those colonies over time by determining colony forming units. In this experiment, *aprE-gfp* reporter strains were mixed with competitors at the ratio of 1:1. In the colonies of *aprE-gfp* and wild-type cells, the population ratio of the *aprE-gfp* cells did not change drastically from the initial ratio by 72 h after inoculation (Fig 10B). By contrast, in the colonies of P_*aprE*_*-gfp* and Δ*yitR-yitM* Δ*sigW* cells, a drastic increase in the population ratio of the *aprE-gfp* cells was observed 24 h after inoculation, and its ratio increased to 93% 72 h after inoculation. In colonies of P_*aprE*_*-gfp* Δ*yitR-yitM* Δ*sigW* and wild-type cells, the population ratio of the P_*aprE*_*-gfp* Δ*yitR-yitM* Δ*sigW* cells decreased to 4% by 72 h after inoculation. However, such a decrease was not observed in colonies of P_*aprE*_*-gfp* Δ*yitR-yitM* Δ*sigW and* Δ*yitR-yitM* Δ*sigW* cells. These results demonstrate that the YIT toxin can mediate intercellular competition within biofilms. In conclusion, we propose that the YIT toxin functions within *B*. *subtilis* biofilms without being obstructed by the biofilm matrix polymers with the assistance of NprB, thus protecting the biofilms from YIT toxin-sensitive unfavorable competitors.

### The variety of SDP toxin homologs

Genome comparison revealed that *sdpABC* homologs are widely conserved among *B*. *subtilis* strains (S1 Table). *sdpABC* homologs, including *sdpABC* itself and *yitPOM*, can be classified into five groups based on their genome positions and SdpC homolog sequences (S1 Table, S4 and S5 Figs). Many *B*. *subtilis* strains have one or two *sdpABC* homologs, and *yitPOM* appears to be more widely conserved among *B*. *subtilis* strains than the other *sdpABC* homologs (S1 Table). Homologous genes encoding membrane proteins are found downstream of *sdpABC* homologs 2 and 4 (S4 Fig). The *sdpIR* homolog are found upstream of the *sdpABC* homolog 3. Some strains possess only *sdpRI* homologs in the *sdpABC* homolog 3 locus. These observations suggest functional similarities between the *sdpABC* homolog 2 and the *sdpABC* homolog 4 and between *sdpABC* and the *sdpABC* homolog 3. However, each group of SdpC homologs has a unique C-terminal hydrophobic domain, the sequence of which differs from those of the others (S5 Fig); therefore, each group of SdpC homolog-derived toxins may have different sequences. If the differences in these sequences impart their functional differences, as observed for SdpC and YitM, then SdpC homolog-derived toxins are likely to play more diverse roles.

## Discussion

Biofilms often contain a high-density bacterial community consisting of a mixture of various species, and under these conditions, the bacteria compete with their own siblings and other species for limited space and nutrients. Since cells in biofilms exhibit increased antibiotic tolerance or resistance, the functions of antibiotics in the competition between biofilm bacteria remain unclear. In this study, we demonstrated that *B*. *subtilis* produces a biofilm-associated toxin, and that this toxin can attack toxin-sensitive cells within the biofilm by passing through the protective barriers of the biofilm with assistance from an endogenously produced extracellular protease.

The *yitPOM* operon is a paralog of the *sdpABC* operon; however, these operons are under different control and play distinct roles. Transcription of *sdpABC* is induced by low levels of Spo0A-P [44]. Given that low levels of Spo0A-P also induce the expression of biofilm matrix synthesis genes including the *eps* operon [44, 45], it is likely that the SDP toxin is induced during the early phase of biofilm formation. By contrast, *yitPOM* is induced by DegS-DegU in biofilms. Induction of these operons had different effects on *B*. *subtilis* cells lacking genes whose products confer resistance to the SDP and YIT toxins, i.e., *sdpABC* induction prevented colony formation independently of the medium conditions, whereas *yitPOM* specifically inhibited biofilm formation. Like SdpC, YitM contains a C-terminal hydrophobic domain that seems to be processed to produce the YIT toxin. The hydrophobic nature of the YIT toxin probably enables the YIT toxin to penetrate bacterial membrane, as observed for the SDP toxin. However, the sequence of the hydrophobic domain of YitM is different from that of SdpC. We assume that this difference contributes to the differences in the roles of these toxins. The genomes of many *B*. *subtilis* strains have both the *sdpABC* and *yitPOM* operons (S1 Table), suggesting that having both *sdpABC* and *yitPOM* may provide survival advantages in the environment.

Overproduction of biofilm matrix polymers interfered with the function of the SDP toxin but not with that of the YIT toxin, suggesting that the YIT toxin has a mechanism to pass through the layers of the biofilm matrix polymers, and we showed that this mechanism involves the extracellular neutral protease NprB. The YIT toxin could not inhibit biofilm formation in the absence of NprB even though the Δ*nprB* mutation increased the production of the YIT toxin. The requirement of NprB was eliminated by Δ*eps* and Δ*bslA* mutations, either of which impairs biofilm matrix formation and, thus, biofilm formation [24, 28]. Cells in biofilms are encased in the biofilm matrix, which functions as a physical and chemical barrier against antibiotics by limiting their penetration [11–16]. Our results suggest that similar mechanisms might contribute to resistance to the YIT toxin in *B*. *subtilis* biofilms and that the extracellular protease NprB is required for the YIT toxin to pass through these defense barriers. The Δ*nprB* mutation had no significant effect on the composition of the extracellular and cell surface-associated proteins of biofilms nor on biofilm formation. We speculate that NprB may degrades the exopolysaccharides- or BslA polymer-associated protein that interacts with the YIT toxin, or that NprB may digest the YIT toxin smaller. By either or both actions, NprB may enable the YIT toxin molecules to pass through the layers of the biofilm matrix polymers. Moreover, we showed that the Δ*nprB* mutation increased activity of the YIT toxin, suggesting the possibility that the YIT toxin is a substrate for NprB protease. We speculate that NprB may also play a role in controlling YIT toxin levels in biofilms to avoid self-intoxication.

If NprB mediates structural changes in biofilms, that can increase the risk that biofilms become susceptible to antibiotics produced by other bacteria. However, the capability of the YIT toxin to attack sensitive cells within biofilms must be important for maintaining biofilm communities. Biofilms often consist of multiple types and multiple species of bacterial cells, some of which exploit others as free-loaders or cheaters that do not produce biofilm matrix polymers and other public goods [57]. The production of biofilm matrix polymers is metabolically costly; however, biofilm matrix polymers are extracellular products that are accessible even to non-producing cheater cells from which they receive protection [57]. An increase in the number of cheater cells, therefore, can disturb the cooperative relationships within biofilm communities and lead to instability within biofilm communities. The production of antibiotics that can diffuse through the biofilm offers the great advantage of being able to eliminate cheater cells and other unfavorable competitors present in the biofilm. Further work is required to determine which types of *B*. *subtilis* cells or what bacterial species are susceptible to the YIT toxin.

Previous studies showed that positively charged antibiotics interact with negatively charged matrix components, such as extracellular DNA and exopolysaccharides, and impede their penetration into biofilms [12, 15]. The SDP toxin contains two positively charged amino acid residues, whereas the hydrophobic region of YitM contains four negatively charged amino acid residues but no positively charged amino acid residues. These observations suggest that both NprB function and the amino acid sequence of the YIT toxin may be important for the ability of the YIT toxin to pass through the layers of biofilm matrix polymers; however, we have not yet determined the relevant sequence of the YIT toxin.

Bacterial competition is mediated by multiple factors [58]. In addition to the YIT toxin, DegS-DegU directly or indirectly induces non-ribosomally synthesized peptide antibiotics, e.g., bacilysin, fengycin, iturin, difficidin, and bacillomycin, although the repertoire of antibiotic synthesis genes differs from strain to strain and no *B*. *subtilis* strain produces all of these [59, 60, 61, 62]. DegS-DegU also induces a wide array of extracellular degradative enzymes, including six extracellular proteases [47 and references therein]. Although these proteases have been thought to play roles in nutrient acquisition from the surrounding environment, our results suggest that these degradative enzymes may also play roles in competition within biofilms. Indeed, several proteases were previously shown to disrupt biofilms of heterologous bacteria by degrading critical protein components in biofilms [63, 64, 65, 66]. Simultaneously producing multiple antibiotics and degradative enzymes within biofilms affords *B*. *subtilis* the ability to attack competitor cells protected by their own biofilm matrixes and to exclude them from biofilm communities. We expect that antibiotics and degradative enzymes cooperate extensively in *B*. *subtilis* biofilms.

The YIT toxin/NprB system seems to have evolved to be specifically adapted to the *B*. *subtilis* biofilm environment. Some bacteria produce specific antibiotics in biofilms [17–21]. Among them, the *Escherichia coli* ROAR029 strain produces the bacteriocin colicin R in biofilms, and colicin R is more active against biofilms than against planktonic cultures, as is the YIT toxin [20]. *Pseudomonas aeruginosa* produces bacteriocins pyocins and can suppress the growth of pyocin-sensitive bacteria in biofilms [21, 22]. Based on our results and these previous observations, we propose that bacteria may have evolved specialized antibiotics that function in biofilms as biofilm-specific competition mechanisms. The properties of these antibiotics may differ from those of conventional antibiotics. Biofilm-associated antibiotics might serve as anti-biofilm agents, especially in combination with degradative enzymes.

## Materials and methods

### Bacterial strains and culture condition

*B*. *subtilis* strain NCIB3610 and its derivatives used in this study are listed in Table 1. Construction of the *B*. *subtilis* mutants is described in S1 File. Primers used for the strain construction are listed in S2 Table. *B*. *subtilis* strains were maintained in LB (LB Lennox; BD Difco, Franklin Lakes, NJ, USA). For colony morphology observation, *B*. *subtilis* strains were grown at 30°C on LB plates overnight. A small single colony was suspended in 100 μl of LB, and 2 μl of the suspension was spotted onto 2×SG [49], MSgg [24], LB, and SMM media [55]. The plates were incubated at 30°C. Colony morphology was observed after 48 h of incubation on 2×SG and LB, after 72 h on SMM, and after 144 h on MSgg. Colony morphology observation was carried out at least three times and typical examples were shown in figures.

**Table 1:** *B*. *subtilis* strains used in this study.

Strain name	Genotypes	References or construction^a^
NCIB3610	prototroph	24
N1285	*amyE*::P_*spac*-hy_-*yitPOM* (*erm*)	This study
N1263	Δ*yitR-yitM*::*tet*	This study
N1286	*amyE*::P_*spac*-hy_-*yitPOM* (*erm*) Δ*yitR-yitM*::*tet*	N1263 → N1285
NTF88	Δ*sigW*::*cat*	67
N1356	*amyE*::P_*spac*-hy_-*yitPOM* (*erm*) Δ*sigW*::*cat*	NTF88 → N1285
N1357	*amyE*::P_*spac*-hy_-*yitPOM* (*erm*) Δ*yitR-yitM*::*tet* Δ*sigW*::*cat*	NTF88 → N1286
N1333	*amyE*::P_*spac*-hy_-*sdpABC* (*erm*)	This study
N1458	Δ*sdpA-sdpR*::*spc*	This study
N1335	*amyE*::P_*spac*-hy_-*sdpABC* (*erm*) Δ*sdpA-sdpR*::*spc*	N1458 → N1333
N1337	*amyE*::P_*spac*-hy_-*sdpABC* (*erm*) Δ*sigW*::*cat*	NTF88 → N1333
N1340	*amyE*::P_*spac*-hy_-*sdpABC* (*erm*) Δ*sdpA-sdpR*::*spc* Δ*sigW*::*cat*	N1458 → N1335
N741	Δ*sdpA-sdpR*::*spc* Δ*yitR-M*::*tet*	N1458 → N1263
N764	Δ*sdpA-sdpR*::*spc* Δ*yitR-M*::*tet* Δ*sigW*::*cat*	NTF88 → N741
N1498	*amyE*::P_*spac*-hy_-*yitQ* (*erm*) Δ*sdpA-sdpR*::*spc* Δ*yitR-M*::*tet* Δ*sigW*::*cat*	*amyE*::P_*spac*-hy_-*yitQ* (*erm*) → N764
N1497	*amyE*::P_*spac*-hy_-*sdpI* (*erm*) Δ*sdpA-sdpR*::*spc* Δ*yitR-M*::*tet* Δ*sigW*::*cat*	*amyE*::P_*spac*-hy_-*sdpI* (*erm*) → N764
N942	*amyE*::P_*spac*-hy_-*yitPOM* (*erm*) Δ*sdpA-sdpR*::*spc* Δ*yitR-yitM*::*tet*	N1285 → N741
N776	*amyE*::P_*spac*-hy_-*sdpABC* (*erm*) Δ*sdpA-sdpR*::*spc* Δ*yitR-yitM*::*tet*	N1333 → N741
NTF28	Δ*degU*::*cat*	62
N1443	pHYG2 (promoter-less *gfp*, *tet*)	pHYG2 → NCIB3610
N1444	pHYG2-*yitP* (P_*yitP*_*-gfp*, *tet*)	pHYG2-*yitP* → NCIB3610
N1446	pHYG2-*nprB* (P_*nprB*_*-gfp*, *tet*)	pHYG2-*nprB* → NCIB3610
N345	Δ*degU*::*kan*	This study
N1515	pHYG2-*yitP* (P_*yitP*_*-gfp*, *tet*) Δ*degU*::*kan*	N345 → N1444
N1516	pHYG2-*nprB* (P_*nprB*_*-gfp*, *tet*) Δ*degU*::*kan*	N345 → N1446
N1382	*amyE*::P_*aprE*_*-gfp* (*cat*)	W740 (*amyE*::P_*aprE*_*-gfp* (*cat*)) [68] → NCIB3610
N1268	Δ*sigW*::*neo*	This study
N355	Δ*epsA-O*::*spc*	This study
N1230	*amyE*::P_*spac*-hy_-*yitPOM* (*erm*) Δ*yitR-yitM*::*tet* Δ*sigW*::*neo*	N1268 → N1286
N1253	*amyE*::P_*spac*-hy_-*yitPOM* (*erm*) Δ*yitR-yitM*::*tet* Δ*sigW*::*neo* Δ*epsA-O*::*spc*	N355 → N1230
N1500	*amyE*::P_*spac*-hy_-*yitPOM* (*erm*) Δ*yitR-yitM*::*tet* Δ*sigW*::*neo* Δ*tapA-tasA*::*cat*	N11 (Δ*tapA-tasA*::*cat*) [62] → N1230
N1255	*amyE*::P_*spac*-hy_-*yitPOM* (*erm*) Δ*yitR-yitM*::*tet* Δ*bslA*::*spc*	N254 (Δ*bslA*::*spc*) [28] → N1230
N924	Δ*nprB-yitM*::*tet*	This study
N1290	*amyE*::P_*spac*-hy_-*yitPOM* (*erm*) Δ*nprB-yitM*::*tet*	N924 → N1285
N1238	*amyE*::P_*spac*-hy_-*yitPOM* (*erm*) Δ*nprB-yitM*::*tet* Δ*sigW*::*neo*	N1268 →N1290
N1293	*amyE*::P_*spac*-hy_-*yitPOM* (*erm*) Δ*nprB-yitM*::*tet* Δ*sigW*::*neo* Δ*epsA-O*::*spc*	N355 →N1238
N1503	*amyE*::P_*spac*-hy_-*yitPOM* (*erm*) Δ*nprB-yitM*::*tet* Δ*sigW*::*neo* Δ*tapA-tasA*::*cat*	N11 (Δ*tapA-tasA*::*cat*) [62] →N1238
N1294	*amyE*::P_*spac*-hy_-*yitPOM* (*erm*) Δ*nprB-yitM*::*tet* Δ*sigW*::*neo* Δ*bslA*::*spc*	N254 (Δ*bslA*::*spc*) [28] →N1238
N1358	*amyE*::P_*spac*-hy_-*yitPOM* (*erm*) Δ*yitR-yitM*::*tet* Δ*sigW*::*neo* Δ*sinR*::*cat*	WTF92 (Δ*sinR*::*cat*) [68] → N1238
N999	*amyE*::P_*spac*-hy_-*sdpABC* (*erm*) Δ*sdpA-sdpR*::*spc* Δ*sigW*::*neo*	N1268 →N1335
N1334	*amyE*::P_*spac*-hy_-*sdpABC* (*erm*) Δ*sdpA-sdpR*::*spc* Δ*sigW*::*neo* Δ*sinR*::*cat*	WTF92 (Δ*sinR*::*cat*) [68] → N999
N1264	*nprB*::*cat*	W115 (*nprB*::*cat*) [47] → NCIB3610
N1287	Δ*yitQ*::*cat*	This study
N1288	Δ*yitQ*::*cat* Δ*sigW*::*neo*	N1268 → N1287
N1234	Δ*yitR-yitM*::*tet* Δ*sigW*::*neo*	N1268 → N1263
N1413	Δ*yitQ*::*spc* Δ*sigW*::*neo amyE*::P_*aprE*_*-gfp* (*cat*)	N1382 → N1288
N1388	Δ*yitR-yitM*::*tet* Δ*sigW*::*neo amyE*::P_*aprE*_*-gfp* (*cat*)	N1382 → N1234

^a^Arrows indicate *B*. *subtilis* transformation: donor strain name → recipient strain name.

### Comparison of genetic organization and protein sequence analysis

A comparison of the genetic organization in different *B*. *subtilis* strains was carried out using the MBGD website (http://mbgd.genome.ad.jp/) [67]. Protein alignments were constructed using the Protein BLAST program on the NCBI website (https://www.nlm.nih.gov/) and GENETYX ver.14 (GENETYX, Tokyo, Japan). Kyte &Doolittle hydropathy plots were constructed using the ExPASy website (https://web.expasy.org/protscale/) with a window size of 19 and default settings (hydropathy scale values of amino acids; A,1.8; R, -4.5; N, -3.5; D,-3.5; C 2.5; Q, -3.5; G, -0.4; H, -3.2; I, 4.5; L, 3.8; K, -3.9; M, 1.9; F, 2.8; P, -1.6;S, -0.8; T, -0.7; W, -1.3; V, 4.2).

### Comparison of growth profiles

*B*. *subtilis* strains grown at 30°C on LB plates overnight were inoculated into 5 ml of 2×SG and were grown at 37°C to the mid-exponential phase with vigorously shaking. These cultures were then added to 50 ml of warm 2×SG in a 500 ml baffled flask to give an OD_600_ of 0.01. These cultures were shaking at 37°C, and OD_600_ of these cultures was measured over time. The experiments were performed at least three times, and the typical results were shown in figures.

### Spot-on-lawn assay

Indicator strains (lawn strains) were grown at 28°C overnight in LB with vigorous shaking. Culture (1 μl) was mixed with 12 ml of 50°C 2×SG 1.2% agar with brief vortexing, and the mixture was immediately poured into a ϕ9 cm plate. The lawn plates were dried for 20 min in a laminar flow cabinet. The strains tested for antibiotic production were grown at 28°C overnight in LB with vigorous shaking. These cultures were diluted 10 times with LB, and 2 μl of the dilutions were spotted onto the dried lawn plates. The plates were incubated at 37°C for 24 to 30 h until halos appeared around the colonies. The experiments were performed at least three times, and the typical example was shown in figures.

### Northern blot analysis

Wild-type and Δ*degU* mutant cells were grown at 37°C in 2×SG with vigorous shaking, and samples were taken from the cultures at various time points for RNA isolation. Total RNA was prepared as previously described [47]. The Northern blot analysis was carried out as previously described [47]. Primers used for RNA probe synthesis are shown in S2 Table.

### Microscopic observation

The strains carrying *gfp* reporters were grown on 2×SG or LB solid medium. The expression of the GFP reporters on the colonies was analyzed with a SZX7 stereomicroscope (Olympus, Tokyo, Japan) equipped with an AdvanCam-E3Rs digital color camera (Advan Vision, Tokyo, Japan). For colony observation, the plates were tilted slightly using a small piece of cardboard (1.5 mm thickness) under the microscope to avoid detecting excitation light reflections on the surfaces of colonies and solid media. However, we could not remove reflected light completely, and some reflected light was observed as blue color signals on GFP images. Images were obtained and processed with AdvanView (Advan Vision) and Photoshop Elements (Adobe, San Jose, CA, USA). The experiments were done at least three times, and the typical examples were shown in figures.

### Flow cytometry analysis

Strains harboring promoter-*gfp* fusions were grown on 2×SG or LB solid medium. A whole single colony was scraped from the surface of the solid medium and suspended in 1 ml of PBS buffer. Cells in the biofilms were then dispersed by repetitive pipetting and were fixed in 4% paraformaldehyde for 7 min [69]. Prior to flow cytometry analysis, the cells were subjected to mild sonication [69]. Single-cell fluorescence was measured on an Accuri C6 flow cytometer (BD Biosciences, Franklin Lakes, NJ, USA). The number of recorded events was 50,000. The experiments were done twice, and the typical examples were shown in figures.

### Competition assay

Strains were grown at 28°C overnight in LB with vigorous shaking. Cultures of the strains used for the assay contained 3.0 × 10^8^ cells/ml on average. The cultures were diluted 100-fold in LB, and two dilutions were mixed at the indicated ratios. Aliquots (2 μl each) of the mixtures were spotted onto 2×SG solid medium, and the plates were incubated at 30°C for 48 h. GFP fluorescence on the colonies was analyzed as described above. The experiments were done three times, and the typical examples were shown in figures. The population ratios of *aprE-gfp* cells in these colonies were also analyzed by determining the number of cells per colonies. A whole single colony was scraped with an inoculation loop and was dissolved in 1 ml of LB in a test tube. After 10 times pipetting, the cell suspension was left for a while until the unsolved cell aggregates went down to the bottom of the tube. The dissolved cells were then serially diluted with LB, and these dilutions were plated on LB or LB plus chloramphenicol (Cm). Since *aprE-gfp* cells exhibited Cm^r^, the population ratio of *aprE-gfp* cells was calculated as Cm^r^ CFUs/ total CFUs. Each population ratio was the average of 4 independent colony measurements.

## Supporting information

S1 Fig The colony morphology of P_*spac*-hy_-*yitPOM* mutants on MSgg medium with 1 mM IPTG. The strains were grown at 30°C on MSgg. Scale bar, 2 mm.
(TIF)

S2 Fig The growth profile of the P_*spac*-hy_-*yitPOM* Δ*yitR*-*yitM* Δ*sigW* mutant in LB with or without 1 mM IPTG. (TIF)

S3 Fig The production of the YIT toxin. Δ*yitR-yitM* Δ*sigW* and Δ*nprB-yitM* Δ*sigW* cells were added to 1.2% 2×SG agar with or without 1000 μM IPTG, and the mixtures were poured into plates. P_*spac*-hy_-*yitPOM* Δ*yitR-yitM* and P_*spac*-hy_-*yitPOM* Δ*nprB-yitM* cells were spotted on the lawn plates. The plates were incubated at 37°C for 24 h.
(TIF)

S4 Fig Comparison of the genetic organization of the *sdpABC* homologs in different *B*. *subtilis* strains. The genetic organization of *sdpABC* and *sdpABC* homologs in the indicated *B*. *subtilis* strains was compared with that of the corresponding locus in strains that do not have *sdpABC* or *sdpABC* homologs. Homologous genes are shown by patterned boxes of the same color. Strain names are shown to the right of the gene maps.
(TIF)

S5 Fig Comparison of SdpC homologs. (A) The alignment of SdpC homologs. The sequences of SdpC homolog 1 (YitM), homolog 2, homolog 3, and homolog 4 are derived from *B*. *subtilis* strains NCIB3610, ATCC13952, BEST195, and OH131.1, respectively. The signal sequences and the SDP toxin sequence are shown in blue and red, respectively. Hydrophobic amino acid residues in the C-terminal regions of SdpC homologs are shown in green. Identical and similar amino acids among all of the homologs are indicated by asterisks and dots, respectively. (B) Hydropathy plots of SdpC homologs. The plots were constructed using the ExPASy website (https://web.expasy.org/protscale/) with a window size of 19.
(TIF)

S1 Table The distribution of *sdpABC* and its homologs in selected *B*. *subtilis strains*. (DOCX)

S2 Table Primers used in this study. (DOCX)

S1 File Construction of *B*. *subtilis* mutants. (PDF)

## References

[1] Hibbing ME, Fuqua C, Parsek MR, Peterson SB. Bacterial competition: surviving and thriving in the microbial jungle. Nat Rev Microbiol. 2010 1;8(1):15–25. 10.1038/nrmicro2259 19946288 PMC2879262

[2] Raaijmakers JM, Mazzola M. Diversity and natural functions of antibiotics produced by beneficial and plant pathogenic bacteria. Annu Rev Phytopathol. 2012;50:403–24. 10.1146/annurev-phyto-081211-172908 22681451

[3] Liu G, Chater KF, Chandra G, Niu G, Tan H. Molecular regulation of antibiotic biosynthesis in streptomyces. Microbiol Mol Biol Rev. 2013 3;77(1):112–43. 10.1128/MMBR.00054-12 23471619 PMC3591988

[4] Stein T. *Bacillus subtilis* antibiotics: structures, syntheses and specific functions. Mol Microbiol. 2005 5;56(4):845–57. 10.1111/j.1365-2958.2005.04587.x 15853875

[5] Davies D. Understanding biofilm resistance to antibacterial agents. Nat Rev Drug Discov. 2003 2;2(2):114–22. 10.1038/nrd1008 12563302

[6] Høiby N, Bjarnsholt T, Givskov M, Molin S, Ciofu O. Antibiotic resistance of bacterial biofilms. Int J Antimicrob Agents. 2010 4;35(4):322–32. 10.1016/j.ijantimicag.2009.12.011 20149602

[7] Ratcliff WC, Denison RF. Alternative actions for antibiotics. Science. 2011 4 29;332(6029):547–8. 10.1126/science.1205970 21527704

[8] Branda SS, Vik S, Friedman L, Kolter R. Biofilms: the matrix revisited. Trends Microbiol. 2005 1;13(1):20–6. 15639628 10.1016/j.tim.2004.11.006

[9] Flemming HC, Wingender J. The biofilm matrix. Nat Rev Microbiol. 2010 9;8(9):623–33. 10.1038/nrmicro2415 20676145

[10] Rendueles O, Ghigo JM. Multi-species biofilms: how to avoid unfriendly neighbors. FEMS Microbiol Rev. 2012 9;36(5):972–89. 10.1111/j.1574-6976.2012.00328.x 22273363

[11] Mulcahy H, Charron-Mazenod L, Lewenza S. Extracellular DNA chelates cations and induces antibiotic resistance in *Pseudomonas aeruginosa* biofilms. PLoS Pathog. 2008 11;4(11):e1000213 10.1371/journal.ppat.1000213 19023416 PMC2581603

[12] Billings N, Millan M, Caldara M, Rusconi R, Tarasova Y, Stocker R, Ribbeck K. The extracellular matrix Component Psl provides fast-acting antibiotic defense in *Pseudomonas aeruginosa* biofilms. PLoS Pathog. 2013;9(8):e1003526 10.1371/journal.ppat.1003526 23950711 PMC3738486

[13] Tseng BS, Zhang W, Harrison JJ, Quach TP, Song JL, Penterman J, Singh PK, Chopp DL, Packman AI, Parsek MR. The extracellular matrix protects *Pseudomonas aeruginosa* biofilms by limiting the penetration of tobramycin. Environ Microbiol. 2013 10;15(10):2865–78. 10.1111/1462-2920.12155 23751003 PMC4045617

[14] Toska J, Ho BT, Mekalanos JJ. Exopolysaccharide protects *Vibrio cholerae* from exogenous attacks by the type 6 secretion system. Proc Natl Acad Sci U S A. 2018 7 31;115(31):7997–8002. 10.1073/pnas.1808469115 30021850 PMC6077691

[15] Doroshenko N, Tseng BS, Howlin RP, Deacon J, Wharton JA, Thurner PJ, Gilmore BF, Parsek MR, Stoodley P. Extracellular DNA impedes the transport of vancomycin in *Staphylococcus epidermidis* biofilms preexposed to subinhibitory concentrations of vancomycin. Antimicrob Agents Chemother. 2014 12;58(12):7273–82. 10.1128/AAC.03132-14 25267673 PMC4249571

[16] Singh R, Sahore S, Kaur P, Rani A, Ray P. Penetration barrier contributes to bacterial biofilm-associated resistance against only select antibiotics, and exhibits genus-, strain- and antibiotic-specific differences. Pathog Dis. 2016 8;74(6). pii: ftw056 10.1093/femspd/ftw056 27402781

[17] Yan L, Boyd KG, Adams DR, Burgess JG. Biofilm-specific cross-species induction of antimicrobial compounds in *bacilli*. Appl Environ Microbiol. 2003 7;69(7):3719–27. 10.1128/AEM.69.7.3719-3727.2003 12839737 PMC165162

[18] Kreth J, Merritt J, Bordador C, Shi W, Qi F. Transcriptional analysis of mutacin I (*mutA*) gene expression in planktonic and biofilm cells of *Streptococcus mutans* using fluorescent protein and glucuronidase reporters. Oral Microbiol Immunol. 2004 8;19(4):252–6. 15209996 10.1111/j.1399-302X.2004.00148.x

[19] Nandi M, Berry C, Brassinga AK, Belmonte MF, Fernando WG, Loewen PC, de Kievit TR. *Pseudomonas brassicacearum* strain DF41 kills *Caenorhabditis elegans* through biofilm-dependent and biofilm-independent mechanisms. Appl Environ Microbiol. 2016 12;82(23):6889–6898. 10.1128/AEM.02199-16 27637885 PMC5103088

[20] Rendueles O, Beloin C, Latour-Lambert P, Ghigo JM. A new biofilm-associated colicin with increased efficiency against biofilm bacteria. ISME J. 2014 6;8(6):1275–88. 10.1038/ismej.2013.238 24451204 PMC4030232

[21] Waite RD, Curtis MA. *Pseudomonas aeruginosa* PAO1 pyocin production affects population dynamics within mixed-culture biofilms. J Bacteriol. 2009 2;191(4):1349–54. 10.1128/JB.01458-08 19060137 PMC2631993

[22] Oluyombo O, Penfold CN, Diggle SP. Competition in biofilms between cystic fibrosis isolates of *Pseudomonas aeruginosa* is shaped by R-pyocins. MBio. 2019 1 29;10(1). pii: e01828–18. 10.1128/mBio.01828-18 30696740 PMC6355985

[23] Anderson MS, Garcia EC, Cotter PA. Kind discrimination and competitive exclusion mediated by contact-dependent growth inhibition systems shape biofilm community structure. PLoS Pathog. 2014 4 17;10(4):e1004076 10.1371/journal.ppat.1004076 24743836 PMC3990724

[24] Branda SS, González-Pastor JE, Ben-Yehuda S, Losick R, Kolter R. Fruiting body formation by *Bacillus subtilis*. Proc Natl Acad Sci U S A. 2001 9 25;98(20):11621–6. 11572999 10.1073/pnas.191384198 PMC58779

[25] Branda SS, González-Pastor JE, Dervyn E, Ehrlich SD, Losick R, Kolter R. 2004. Genes involved in formation of structured multicellular communities by *Bacillus subtilis*. J Bacteriol. 2004 6;186(12):3970–9. 10.1128/JB.186.12.3970-3979.2004 15175311 PMC419949

[26] Branda SS, Chu F, Kearns DB, Losick R, Kolter R. A major protein component of the *Bacillus subtilis* biofilm matrix. Mol Microbiol. 2006 2;59(4):1229–38. 10.1111/j.1365-2958.2005.05020.x 16430696

[27] Romero D, Aguilar C, Losick R, Kolter R. Amyloid fibers provide structural integrity to *Bacillus subtilis* biofilms. Proc Natl Acad Sci U S A. 2010 2 2;107(5):2230–4 10.1073/pnas.0910560107 20080671 PMC2836674

[28] Kobayashi K, Iwano M. BslA(YuaB) forms a hydrophobic layer on the surface of *Bacillus subtilis* biofilms. Mol Microbiol. 2012 7;85(1):51–66 10.1111/j.1365-2958.2012.08094.x 22571672

[29] Hobley L, Ostrowski A, Rao FV, Bromley KM, Porter M, Prescott AR, MacPhee CE, van Aalten DM, Stanley-Wall NR. BslA is a self-assembling bacterial hydrophobin that coats the *Bacillus subtilis* biofilm. Proc Natl Acad Sci U S A. 2013 8 13;110(33):13600–5. 10.1073/pnas.1306390110 23904481 PMC3746881

[30] Hamon MA, Stanley NR, Britton RA, Grossman AD, Lazazzera BA. Identification of AbrB-regulated genes involved in biofilm formation by *Bacillus subtilis*. Mol Microbiol. 2004 5;52(3):847–60. 10.1111/j.1365-2958.2004.04023.x 15101989 PMC1409746

[31] Kearns DB, Chu F, Branda SS, Kolter R, Losick R. A master regulator for biofilm formation by *Bacillus subtilis*. Mol Microbiol. 2005 2;55(3):739–49. 10.1111/j.1365-2958.2004.04440.x 15661000

[32] Chu F, Kearns DB, Branda SS, Kolter R, Losick R. Targets of the master regulator of biofilm formation in *Bacillus subtilis*. Mol Microbiol. 2006 2;59(4):1216–28. 10.1111/j.1365-2958.2005.05019.x 16430695

[33] Verhamme DT, Murray EJ, Stanley-Wall NR. DegU and Spo0A jointly control transcription of two loci required for complex colony development by *Bacillus subtilis*. J Bacteriol. 2009 1;191(1):100–8. 10.1128/JB.01236-08 18978066 PMC2612447

[34] López D, Fischbach MA, Chu F, Losick R, Kolter R. Structurally diverse natural products that cause potassium leakage trigger multicellularity in *Bacillus subtilis*. Proc Natl Acad Sci U S A. 2009 1 6;106(1):280–5. 10.1073/pnas.0810940106 19114652 PMC2629187

[35] Mielich-Süss B, Lopez D. Molecular mechanisms involved in *Bacillus subtilis* biofilm formation. Environ Microbiol. 2015 3;17(3):555–65. 24909922 10.1111/1462-2920.12527 PMC4188541

[36] Straight PD, Fischbach MA, Walsh CT, Rudner DZ, Kolter R. A singular enzymatic megacomplex from *Bacillus subtilis*. Proc Natl Acad Sci U S A. 2007 1 2;104(1):305–10. 10.1073/pnas.0609073103 17190806 PMC1765455

[37] Abriouel H, Franz CM, Ben Omar N, Gálvez A. Diversity and applications of *Bacillus* bacteriocins. FEMS Microbiol Rev. 2011 1;35(1):201–32. 10.1111/j.1574-6976.2010.00244.x 20695901

[38] Koskiniemi S, Lamoureux JG, Nikolakakis KC, t'Kint de Roodenbeke C, Kaplan MD, Low DA, Hayes CS. Rhs proteins from diverse bacteria mediate intercellular competition. Proc Natl Acad Sci U S A. 2013 4 23;110(17):7032–7. 10.1073/pnas.1300627110 23572593 PMC3637788

[39] Liu WT, Yang YL, Xu Y, Lamsa A, Haste NM, Yang JY, Ng J, Gonzalez D, Ellermeier CD, Straight PD, Pevzner PA, Pogliano J, Nizet V, Pogliano K, Dorrestein PC. Imaging mass spectrometry of intraspecies metabolic exchange revealed the cannibalistic factors of *Bacillus subtilis*. Proc Natl Acad Sci U S A. 2010 9 14;107(37):16286–90. 10.1073/pnas.1008368107 20805502 PMC2941286

[40] González-Pastor JE, Hobbs EC, Losick R. Cannibalism by sporulating bacteria. Science. 2003 7 25;301(5632):510–3 10.1126/science.1086462 12817086

[41] Pérez Morales TG, Ho TD, Liu WT, Dorrestein PC, Ellermeier CD. Production of the cannibalism toxin SDP is a multistep process that requires SdpA and SdpB. J Bacteriol. 2013 7;195(14):3244–51. 10.1128/JB.00407-13 23687264 PMC3697648

[42] Lamsa A, Liu WT, Dorrestein PC, Pogliano K. The *Bacillus subtilis* cannibalism toxin SDP collapses the proton motive force and induces autolysis. Mol Microbiol. 2012 5;84(3):486–500. 10.1111/j.1365-2958.2012.08038.x 22469514 PMC3839633

[43] Ellermeier CD, Hobbs EC, Gonzalez-Pastor JE, Losick R. A three-protein signaling pathway governing immunity to a bacterial cannibalism toxin. Cell. 2006 2 10;124(3):549–59. 10.1016/j.cell.2005.11.041 16469701

[44] Fujita M, Losick R. Evidence that entry into sporulation in *Bacillus subtilis* is governed by a gradual increase in the level and activity of the master regulator Spo0A. Genes Dev. 2005 9 15;19(18):2236–44. 10.1101/gad.1335705 16166384 PMC1221893

[45] López D, Vlamakis H, Losick R, Kolter R. Cannibalism enhances biofilm development in *Bacillus subtilis*. Mol Microbiol. 2009 11;74(3):609–18. 10.1111/j.1365-2958.2009.06882.x 19775247 PMC2983100

[46] Lyons NA, Kraigher B, Stefanic P, Mandic-Mulec I, Kolter R. A Combinatorial Kin Discrimination System in *Bacillus subtilis*. Curr Biol. 2016 3 21;26(6):733–42. 10.1016/j.cub.2016.01.032 26923784 PMC4803606

[47] Kobayashi K. Gradual activation of the response regulator DegU controls serial expression of genes for flagellum formation and biofilm formation in *Bacillus subtilis*. Mol Microbiol. 2007 10;66(2):395–409. 10.1111/j.1365-2958.2007.05923.x 17850253

[48] Verhamme DT, Kiley TB, Stanley-Wall NR. DegU co-ordinates multicellular behaviour exhibited by *Bacillus subtilis*. Mol Microbiol. 2007 7;65(2):554–68. 10.1111/j.1365-2958.2007.05810.x 17590234

[49] Quisel JD, Burkholder WF, Grossman AD. *In vivo* effects of sporulation kinases on mutant Spo0A proteins in *Bacillus subtilis*. J Bacteriol. 2001 11;183(22):6573–8. 10.1128/JB.183.22.6573-6578.2001 11673427 PMC95488

[50] Leighton TJ, Doi RH. The stability of messenger ribonucleic acid during sporulation in *Bacillus subtilis*. J Biol Chem. 1971 5 25;246(10):3189–95. 4995746

[51] Butcher BG, Helmann JD. Identification of *Bacillus subtilis* sigma-dependent genes that provide intrinsic resistance to antimicrobial compounds produced by *Bacilli*. Mol Microbiol. 2006 5;60(3):765–82. 10.1111/j.1365-2958.2006.05131.x 16629676

[52] Cao M, Bernat BA, Wang Z, Armstrong RN, Helmann JD. FosB, a cysteine-dependent fosfomycin resistance protein under the control of sigma(W), an extracytoplasmic-function sigma factor in *Bacillus subtilis*. J Bacteriol. 2001 4;183(7):2380–3. 10.1128/JB.183.7.2380-2383.2001 11244082 PMC95149

[53] Yamada Y, Tikhonova EB, Zgurskaya HI. YknWXYZ is an unusual four-component transporter with a role in protection against sporulation-delaying-protein-induced killing of *Bacillus subtilis*. J Bacteriol. 2012 8;194(16):4386–94. 10.1128/JB.00223-12 22707703 PMC3416220

[54] Huang X, Gaballa A, Cao M, Helmann JD. Identification of target promoters for the *Bacillus subtilis* extracytoplasmic function sigma factor, sigma W. Mol Microbiol. 1999 1;31(1):361–71. 10.1046/j.1365-2958.1999.01180.x 9987136

[55] Marlow VL, Cianfanelli FR, Porter M, Cairns LS, Dale JK, Stanley-Wall NR. The prevalence and origin of exoprotease-producing cells in the *Bacillus subtilis* biofilm. Microbiology. 2014 1;160(Pt 1):56–66. 10.1099/mic.0.072389-0 24149708 PMC3917226

[56] Spizizen J. Transformation of Biochemically Deficient Strains of *Bacillus subtilis* by Deoxyribonucleate. Proc Natl Acad Sci U S A. 1958 10 15;44(10):1072–8. 10.1073/pnas.44.10.1072 16590310 PMC528696

[57] West SA, Diggle SP, Buckling A, Gardner A, Griffin AS. The Social lives of microbes. Ann Rev Ecol Evol Syst. 2007;38:53–77.

[58] Lyons NA, Kraigher B, Stefanic P, Mandic-Mulec I, Kolter R. A Combinatorial Kin Discrimination System in *Bacillus subtilis*. Curr Biol. 2016 3 21;26(6):733–42. 10.1016/j.cub.2016.01.032 26923784 PMC4803606

[59] Tsuge K, Ano T, Hirai M, Nakamura Y, Shoda M. The genes *degQ*, *pps*, and *lpa-8* (*sfp*) are responsible for conversion of *Bacillus subtilis* 168 to plipastatin production. Antimicrob Agents Chemother. 1999 9;43(9):2183–92. 10471562 10.1128/aac.43.9.2183 PMC89444

[60] Koumoutsi A, Chen XH, Vater J, Borriss R. DegU and YczE positively regulate the synthesis of bacillomycin D by *Bacillus amyloliquefaciens* strain FZB42. Appl Environ Microbiol. 2007 11;73(21):6953–64. 10.1128/AEM.00565-07 17827323 PMC2074971

[61] Chen XH, Scholz R, Borriss M, Junge H, Mögel G, Kunz S, Borriss R. Difficidin and bacilysin produced by plant-associated *Bacillus amyloliquefacien*s are efficient in controlling fire blight disease. J Biotechnol. 2009 3 10;140(1–2):38–44. 10.1016/j.jbiotec.2008.10.015 19061923

[62] Kobayashi K. Plant methyl salicylate induces defense responses in the rhizobacterium *Bacillus subtilis*. Environ Microbiol. 2015 4;17(4):1365–76. 10.1111/1462-2920.12613 25181478

[63] Iwase T, Uehara Y, Shinji H, Tajima A, Seo H, Takada K, Agata T, Mizunoe Y. *Staphylococcus epidermidis* Esp inhibits *Staphylococcus aureus* biofilm formation and nasal colonization. Nature. 2010 5 20;465(7296):346–9. 10.1038/nature09074 20485435

[64] Baidamshina DR, Trizna EY, Holyavka MG, Bogachev MI, Artyukhov VG, Akhatova FS, Rozhina EV, Fakhrullin RF, Kayumov AR. Targeting microbial biofilms using Ficin, a nonspecific plant protease. Sci Rep. 2017 4 7;7:46068 10.1038/srep46068 28387349 PMC5384253

[65] Mitrofanova O, Mardanova A, Evtugyn V, Bogomolnaya L, Sharipova M. Effects of *Bacillus* serine proteases on the bacterial biofilms. Biomed Res Int. 2017;2017:8525912 10.1155/2017/8525912 28904973 PMC5585633

[66] Kumar L, Cox CR, Sarkar SK. Matrix metalloprotease-1 inhibits and disrupts *Enterococcus faecalis* biofilms. PLoS One. 2019 1 11;14(1):e0210218 10.1371/journal.pone.0210218 30633757 PMC6329490

[67] Kobayashi K. *Bacillus subtilis* pellicle formation proceeds through genetically defined morphological changes. J Bacteriol. 2007 7;189(13):4920–31. 10.1128/JB.00157-07 17468240 PMC1913431

[68] Uchiyama I, Mihara M, Nishide H, Chiba H. MBGD update 2015: microbial genome database for flexible ortholog analysis utilizing a diverse set of genomic data. Nucleic Acids Res. 2015 1;43(Database issue):D270–6. 10.1093/nar/gku1152 25398900 PMC4383954

[69] Vlamakis H, Aguilar C, Losick R, Kolter R. Control of cell fate by the formation of an architecturally complex bacterial community. Genes Dev. 2008 4 1;22(7):945–53. 18381896 10.1101/gad.1645008 PMC2279205

