# Supplementary figures and images for: Biofilm-associated toxin and extracellular protease cooperatively suppress competitors in Bacillus subtilis biofilms
Source: PLoS Genet. 2019 Oct 17;15(10):e1008232. doi: 10.1371/journal.pgen.1008232 (PMC6818787; doi:10.1371/journal.pgen.1008232)

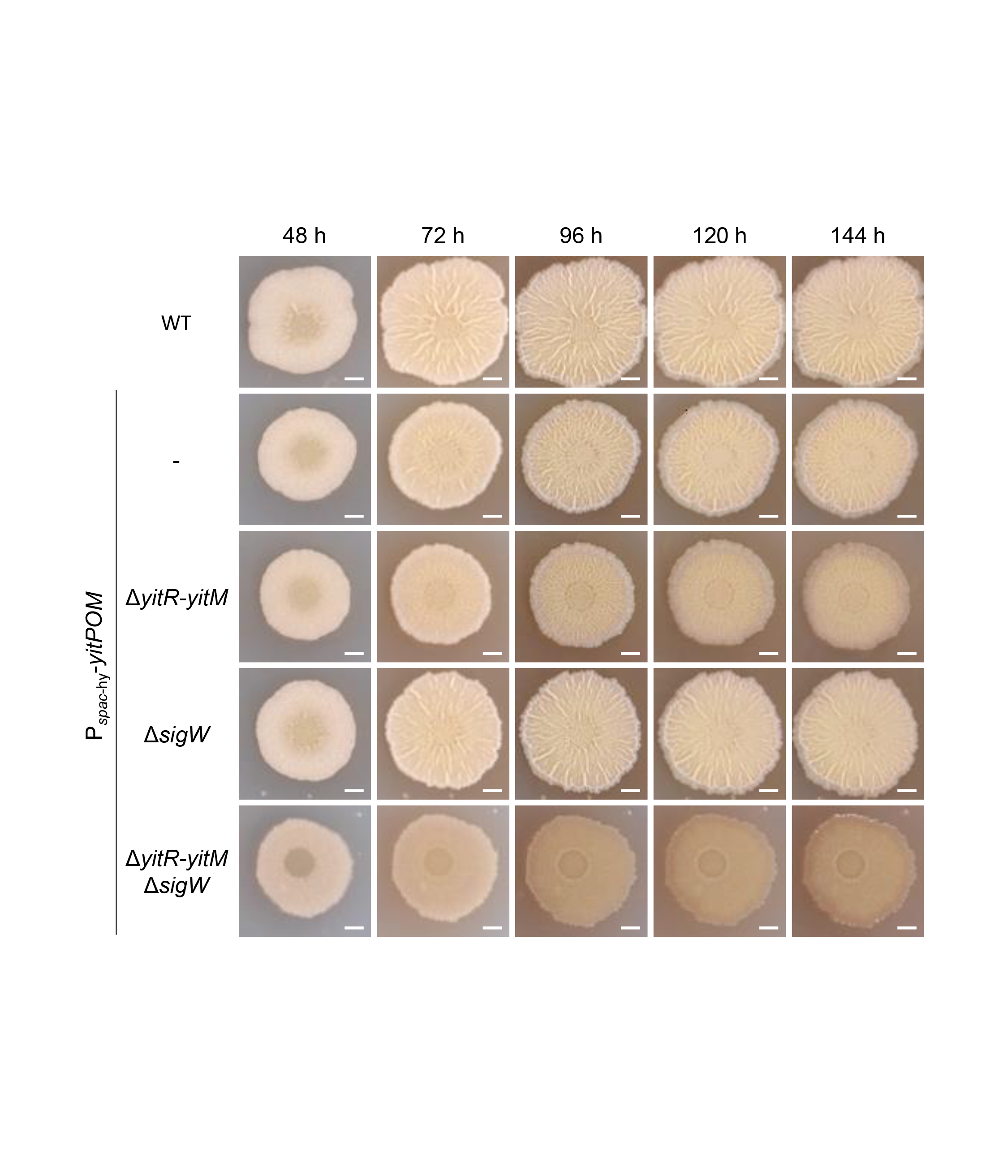

Supplement: S1 Fig — The strains were grown at 30°C on MSgg. Scale bar, 2 mm. (TIF) [file pgen.1008232.s001.tif]

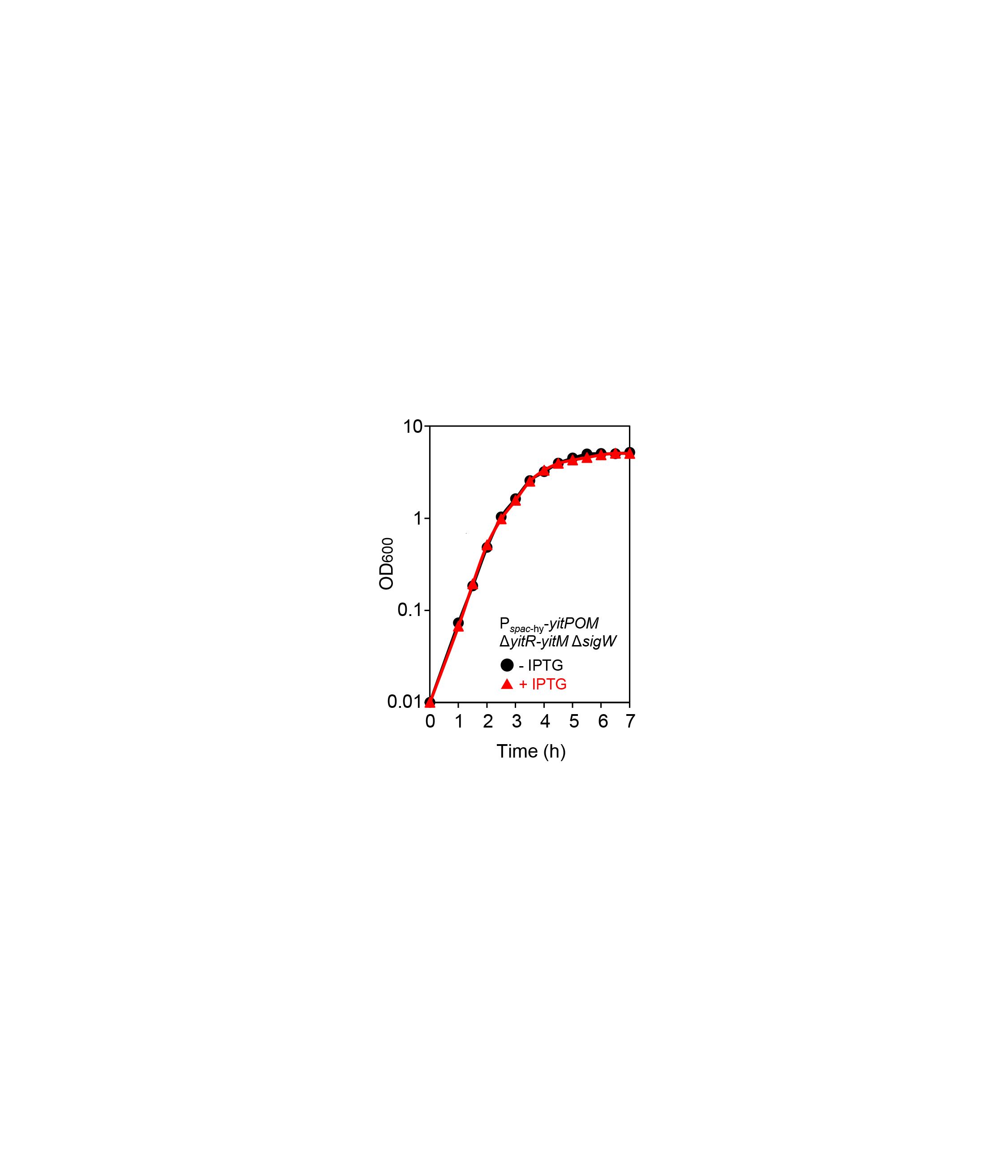

Supplement: S2 Fig — (TIF) [file pgen.1008232.s002.tif]

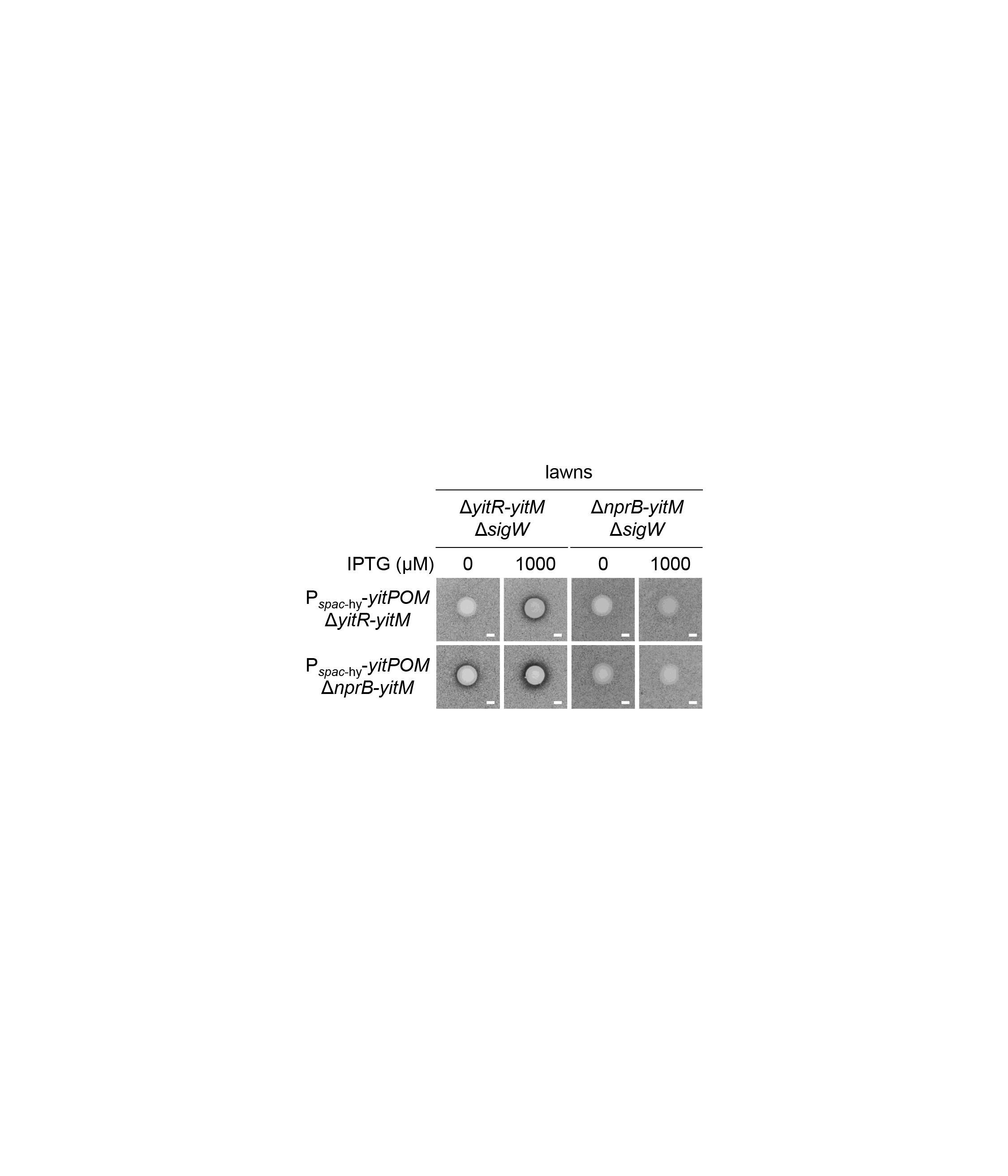

Supplement: S3 Fig — ΔyitR-yitM ΔsigW and ΔnprB-yitM ΔsigW cells were added to 1.2% 2×SG agar with or without 1000 μM IPTG, and the mixtures were poured into plates. Pspac-hy-yitPOM ΔyitR-yitM and Pspac-hy-yitPOM ΔnprB-yitM cells were spotted on the lawn plates. The plates were incubated at 37°C for 24 h. (TIF) [file pgen.1008232.s003.tif]

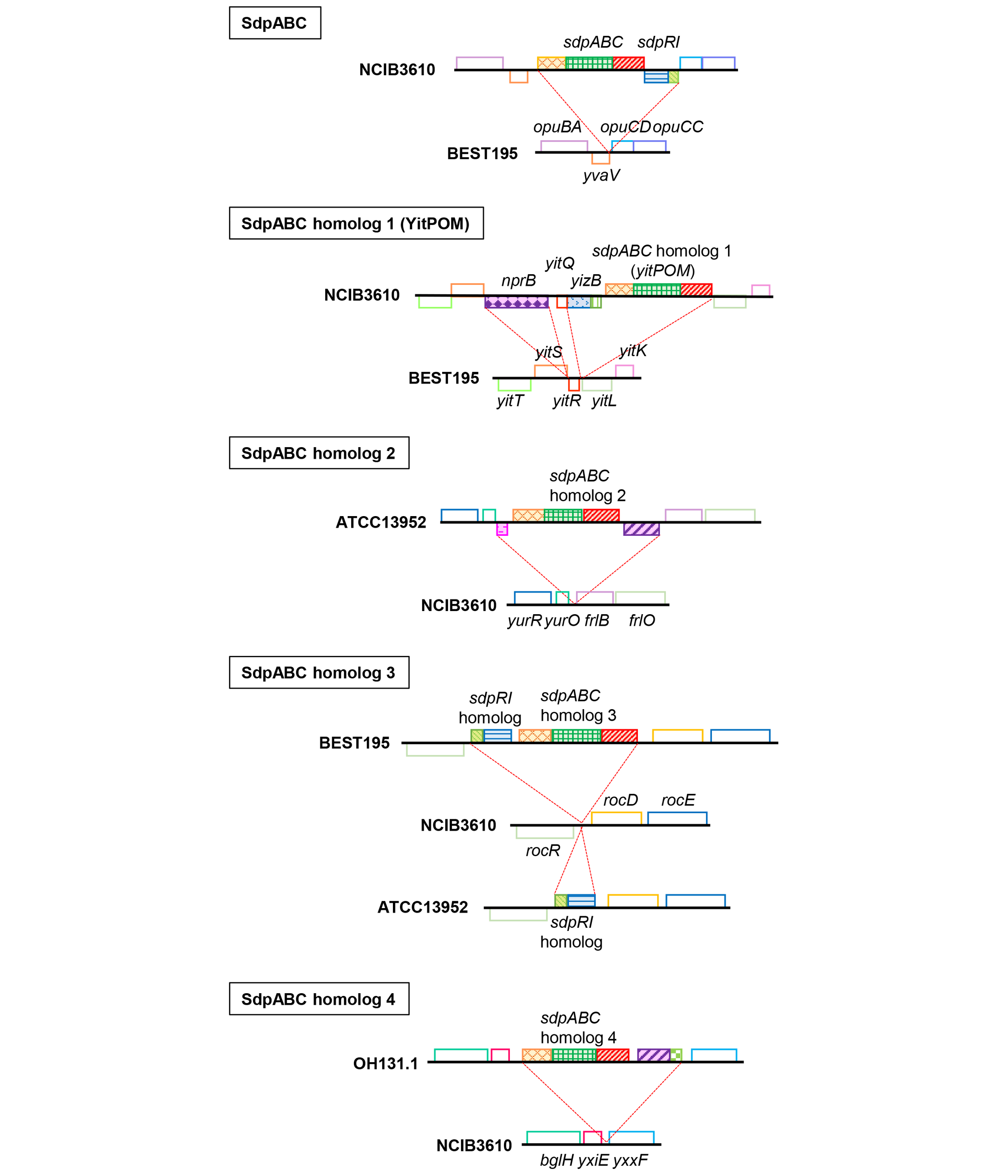

Supplement: S4 Fig — The genetic organization of sdpABC and sdpABC homologs in the indicated B. subtilis strains was compared with that of the corresponding locus in strains that do not have sdpABC or sdpABC homologs. Homologous genes are shown by patterned boxes of the same color. Strain names are shown to the right of the gene maps. (TIF) [file pgen.1008232.s004.tif]

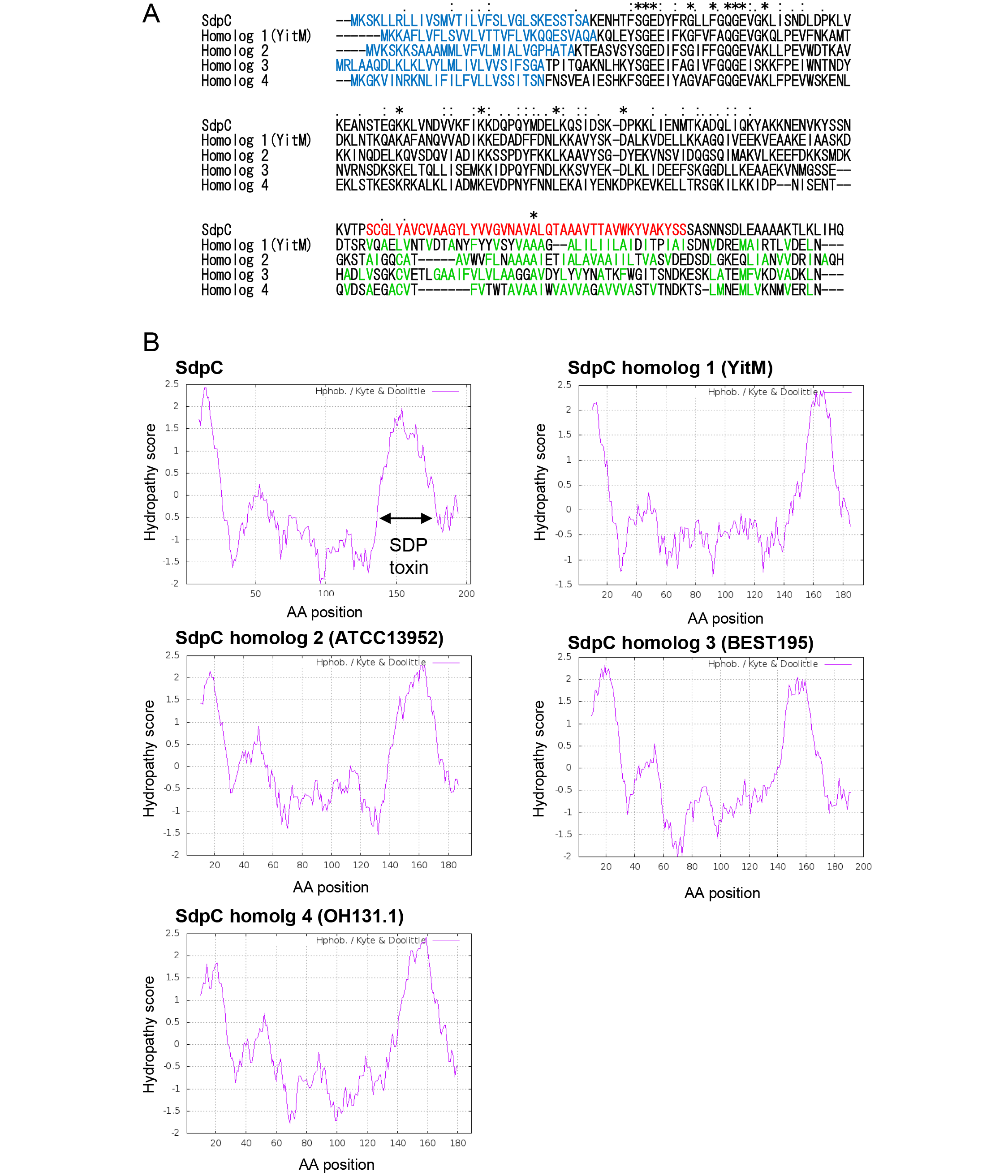

Supplement: S5 Fig — (A) The alignment of SdpC homologs. The sequences of SdpC homolog 1 (YitM), homolog 2, homolog 3, and homolog 4 are derived from B. subtilis strains NCIB3610, ATCC13952, BEST195, and OH131.1, respectively. The signal sequences and the SDP toxin sequence are shown in blue and red, respectively. Hydrophobic amino acid residues in the C-terminal regions of SdpC homologs are shown in green. Identical and similar amino acids among all of the homologs are indicated by asterisks and dots, respectively. (B) Hydropathy plots of SdpC homologs. The plots were constructed using the ExPASy website (https://web.expasy.org/protscale/) with a window size of 19. (TIF) [file pgen.1008232.s005.tif]
