# Supplementary material for: Biofilm-associated toxin and extracellular protease cooperatively suppress competitors in Bacillus subtilis biofilms
Source: PLoS Genet. 2019 Oct 17;15(10):e1008232. doi: 10.1371/journal.pgen.1008232 (PMC6818787; doi:10.1371/journal.pgen.1008232)
Supplement: S1 Table — (DOCX) [file pgen.1008232.s006.docx]

S1 Table. The distribution of *sdpABC* and its homologs in selected *B. subtilis strains*.^a, b^

| ***B. subtilis* strains** | ***sdpABC*** | ***sdpABC* homolog 1**  **(*yitPOM*)** | ***sdpABC* homolog 2** | ***sdpABC* homolog 3** | ***sdpABC* homolog 4** |
| --- | --- | --- | --- | --- | --- |
| subsp. *subtilis* str. NCIB3610 | ● | ● |  |  |  |
| UD1022 | ● | ● |  |  |  |
| SG6 | ● | ● |  |  |  |
| PS832 | ● | ● |  |  |  |
| TO-A JPC | ● | ● |  |  |  |
| BS49 | ● | ● |  |  |  |
| BEST7003 | ● | ● |  |  |  |
| BEST7613 | ● | ● |  |  |  |
| KCTC1028 | ● | ● |  |  |  |
| QB928 | ● | ● |  |  |  |
| subsp. *subtilis* str. 3NA | ● | ● |  |  |  |
| subsp. *subtilis* str. AG1839 | ● | ● |  |  |  |
| subsp. *subtilis* str. BSP1 | ● | ● |  |  |  |
| XF-1 |  | ● |  |  |  |
| BAB-1 |  | ● |  |  |  |
| HJ5 |  | ● |  |  |  |
| Bs916 |  |  | ● |  |  |
| ATCC13952 |  |  | ● |  |  |
| subsp. *natto* BEST195 |  |  |  | ● |  |
| BSn5 |  | ● |  | ● |  |
| subsp. *subtilis* OH131.1 |  | ● |  |  | ● |
| RO-NN-1 |  |  |  |  |  |
| ATCC19217 |  |  |  |  |  |
| B-1 |  |  |  |  |  |
| T30 |  |  |  |  |  |
| subsp. *spizizenii* NRS231 |  |  |  |  |  |
| subsp. *spizizenii* TU-B-10 |  |  |  |  |  |
| subsp. *spizizenii* W23 |  |  |  |  |  |

^a^The *sdpABC* homologs were classified into five groups based on their positions in the genomes and the SdpC homolog sequences (see S5 and S6 Figs).

^b^● represents having the homolog.
