# Supplementary material for: Biofilm-associated toxin and extracellular protease cooperatively suppress competitors in Bacillus subtilis biofilms
Source: PLoS Genet. 2019 Oct 17;15(10):e1008232. doi: 10.1371/journal.pgen.1008232 (PMC6818787; doi:10.1371/journal.pgen.1008232)
Supplement: S2 Table — (DOCX) [file pgen.1008232.s007.docx]

S2 Table. Primers used in this study.

| **Primer** | **Sequence (5’ to 3’)** |
| --- | --- |
| **P*_spac_*_-hy_-*yitPOM*** | |
| YI-*yitP*-F1 | AAGAAGCTTCGATTCTTCAGGTTAAAAGGAGCAG |
| YI-*yitP*-R3 | GCCAGGCAAGGATCCTCAGGAAAATGGATGGACATACGAG |
| **P*_spac_*_-hy_-*sdpABC*** | |
| YI-*sdpA*-F1 | AAGAAGCTTTAGCTTAGAGGAGGTAATCTACATC |
| YI-*sdpA*-R3 | GCCAGGCAAGGATCCCCTCTAAAGTACTTGTAGTGTGTGG |
| **P*_spac_*_-hy_-*yitQ*** | |
| *yitQ*-F1 | AAGAAGCTTGGAAACCCATCAAGCCTTAC |
| *yitQ*-R1 | GGAGGATCCCTTTGATTCTGTCTTGAACG |
| **P*_spac_*_-hy_-*sdpI*** | |
| *sdpI*-F1 | AAGAAGCTTCAAGACTCAATAAATTGGATGC |
| *sdpI*-R1 | GGAGGATCCTTTCCTGACCATTGTATCGG |
| **Δ*yitR-yitM*** | |
| *yitR*-F1 | GTTTTCCTTCATCGCGACCG |
| *yitR*-R1 | GTTATCCGCTCACAATTCATACTGGCATATGGAGCAGG |
| *yitPM*-F2 | CGTCGTGACTGGGAAAACAATCAGAACGCTGGTTGATG |
| *yitPM*-R2 | TATCGAAGGACGCACTAGTC |
| **Δ*nprB-yitM*** | |
| *nprB*-F3-2 | TAATGATCGCCAATGAAGTG |
| *nprB*-R3-2 | GTTATCCGCTCACAATTCCTCGAAGCAATCAGCTTCAG |
| *yitPM*-F2 | CGTCGTGACTGGGAAAACAATCAGAACGCTGGTTGATG |
| *yitPM*-R2 | TATCGAAGGACGCACTAGTC |
| **Δ*yitQ*** | |
| *yitQ*-F3-2 | TGGCTATTTCAGAGGGAGCG |
| *yitQ*-R3 | GTTATCCGCTCACAATTCCATCAGGCATATTCGCCAGC |
| *yitQ*-F4 | CGTCGTGACTGGGAAAACTGCGCTTGTCGTCCTGCTGC |
| *yitR*-F1 | GTTTTCCTTCATCGCGACCG |
| **Δ*sdpA-sdpR*** | |
| YI-*sdpA*-F4 | ATCGCGGAAAACATGCATGC |
| YI-*sdpA*-R4 | GTTATCCGCTCACAATTCGAACAGTGGATTCTGAGGT |
| YI-*sdpA*-F5 | CGTCGTGACTGGGAAAACCTTGTTGGATCTGATATAGC |
| YI-*sdpA*-R5 | CATCGGGACATTTGTCGGTG |
| ***cat*** | |
| pUC-F | GTTTTCCCAGTCACGACG |
| pUC-R | GAATTGTGAGCGGATAAC |
| **pHYG2-*yitP*** | |
| *yitP*-P-F1 | GAAGAATTCATCCAGCGGTCATTAACTTG |
| *yitP*-P-R1 | AAGAAGCTTTAGCGAACCCCAAAGGATTG |
| **pHYG2-*nprB*** | |
| *nprB*-P-F1 | TGGCCAGAATAGCAGGCTACT |
| *nprB*-P-R1 | AAGAAGCTTAACCATTTGGGCCGCTGTGC |
| **Probes for Northern blot** | |
| *yitP*-N-F | TTGGCAGCTTAGGGACAACC |
| *yitP*-N-T7R | TAATACGACTCACTATAGGGCGAATGACGATCAGCGATGACTG |
| *yizB*-N-F | ATCAAGTTAATGACCGCTGG |
| *yizB*-N-T7R | TAATACGACTCACTATAGGGCGAAGCAGATAAACCGCACCGAC |
| *nprB*-N-F | TTGCGCAACTTGACCAAGAC |
| *nprB*-N-T7R | TAATACGACTCACTATAGGGCGAAGGTTTCGTTCAGGTTCTTC |
