# Supplementary material for: Biofilm-associated toxin and extracellular protease cooperatively suppress competitors in Bacillus subtilis biofilms
Source: PLoS Genet. 2019 Oct 17;15(10):e1008232. doi: 10.1371/journal.pgen.1008232 (PMC6818787; doi:10.1371/journal.pgen.1008232)
Supplement: S1 File — (PDF) [file pgen.1008232.s008.pdf]

S1 Appendix

Construction of *B. subtilis* mutants

Because strain NCIB3610 has low transformation ability, mutant alleles were first introduced into the domesticated strain 168 and then transferred to strain NCIB3610 via transformation using genomic DNA [1]. Strain 168 has several mutations that affect biofilm formation. The possible introduction of these unwanted mutations into strain NCIB3610 via transformation using genomic DNA was removed by examining colony morphology of transformants. Strain NCIB3610 was directly transformed with pHYG2 and its derivative plasmids. Primers used in the strain construction are shown in S2 Table. The construction of the Δ*sigW*::*cat*, Δ*spo0A*::*cat*, Δ*degU*::*cat*, Δ*eps*::*spc*, Δ*bslA*::*spc*, Δ*sinR*::*cat*, Δ*nprB*::*cat*, and *amyE*::P_{aprE}-*gfp* strains was described previously [1-4]. The *cat* gene of the Δ*sigW*::*cat* mutant was replaced with *neo* using pCm::Neo [5]. The Δ*degU*::*kan* mutant was constructed using the same primer sets with the Δ*degU*::*cat* mutant [1]. The construction of the other strains is described below.

(1) $P_{spac\text{-}hy}$-*yitPOM*, $P_{spac\text{-}hy}$-*sdpABC*, $P_{spac\text{-}hy}$-*yitQ*, and $P_{spac\text{-}hy}$-*sdpI* strains

The entire region of the *yitPOM* locus, including the SD sequence of *yitP*, was amplified via PCR using the primers YI-*yitP*-F1 and YI-*yitP*-R3. The PCR products were digested with *Hin*dIII and *Bam*HI and then ligated with *Hin*dIII and *Bam*HI-digested pDLT3-Hy, an *amyE* integration vector that contains *cat*, *lacI*, and the *spac*-hy promoter between the *amyE* upstream and downstream sequences. The ligation mixture was used to transform *B. subtili*s strain 168, and Cm^r colonies were selected. Chromosomal DNA samples were prepared from these Cm^r colonies, and transformants carrying $P_{spac\text{-}hy}$-*yitPOM* were screened by PCR. One verified transformant was used as the $P_{spac\text{-}hy}$-*yitPOM* strain. The Cm^r marker of the $P_{spac\text{-}hy}$-*yitPOM* strain was replaced with Em^r using the marker exchange plasmid pCm::Em [5]. The

$P_{spac\text{-hy}}$-*yitPOM* construct was then transferred into NCIB3610. The $P_{spac\text{-hy}}$-*sdpABC*, $P_{spac\text{-hy}}$-*yitQ*, and $P_{spac\text{-hy}}$-*sdpI* strains were constructed via the same procedure using different primers. The entire regions of the *sdpABC*, *yitQ*, and *sdpI* loci were amplified via PCR using the primer pairs YI-*yitP*-F1/YI-*yitP*-R3, *yitQ*-F1/*yitQ*-R1, and *sdpI*-F1/*sdpI*-R1, respectively.

(2) Deletion mutants

Deletion of *yitR-yitM* was carried out using an overlap-extension PCR technique. A *cat* cassette was amplified from pCBB31 [1] via PCR using the pUC-R and pUC-F primers (S2 Table). The upstream region of *yitR* and the downstream region of *yitM* were amplified via PCR using the primer pairs *yitR*-F1/*yitR*-R1 and *yitPM*-F2/*yitPM*-R2, respectively. The 5' sequences of *yitR*-R1 and *yitPM*-F2 were complementary to the sequences of pUC-R and pUC-F, respectively. To connect the three PCR fragments, all three were mixed and used as a template for a second round of PCR using primers *spx*-F1 and *spx*-R2. The resultant PCR products were used for transformation of strain 168. Deletion mutants of *nprB-yitM*, *yitQ*, and *sdpA-sdpR* were constructed via the same procedure using different primer sets, i.e., Δ*yitR-yitM*, *yitR*-F1/*yitR*-R1 and *yitPM*-F2/*yitPM*-R2; Δ*nprB-yitM*, *nprB*-F3-2/*nprB*-R3-2 and *yitPM*-F2/*yitPM*-R2; Δ*yitQ*, *yitQ*-F3-2/*yitQ*-R3 and *yitQ*-F4/*yitR*-F1; and Δ*sdpA-sdpR*, YI-*sdpA*-F4/YI-*sdpA*-R4 and YI-*sdpA*-F5/YI-*sdpA*-R5. These deletions were then transferred to NCIB3610 and other strains.

(3) P_{yitP}-*gfp* and P_{nprB}-*gfp*

yitP and *nprB* promoter regions were amplified via PCR using primer sets, the *yitP*-P-F1/*yitP*-P-R1 and *nprB*-P-F1/*nprB*-P-R1primers, respectively. The PCR products were digested with *Eco*RI and *Hin*dIII and ligated with *Eco*RI and *Hin*dIII-digested plasmid pHYG2, which is a derivative of the *E. coli* and *B. subtilis* shuttle plasmid pHY300 PLK [6] containing a promoterless *gfp* gene. The ligation mixture was used to transform *E. coli JM105* to obtain pHYG2-*yitP* and pHYG2-*nprB*. These plasmids were used to transform strain

NCIB3610.

S1 Appendix References

1. Kobayashi K. *Bacillus subtilis* pellicle formation proceeds through genetically defined morphological changes. J Bacteriol. 2007 Jul;189(13):4920-31.
2. Kobayashi K. Gradual activation of the response regulator DegU controls serial expression of genes for flagellum formation and biofilm formation in *Bacillus subtilis*. Mol Microbiol. 2007 Oct;66(2):395-409.
3. Kobayashi K, Iwano M. BslA(YuaB) forms a hydrophobic layer on the surface of *Bacillus subtilis* biofilms. Mol Microbiol. 2012 Jul;85(1):51-66.
4. Kobayashi K. Plant methyl salicylate induces defense responses in the rhizobacterium *Bacillus subtilis*. Environ Microbiol. 2015 Apr;17(4):1365-76.
5. Steinmetz M, Richter R. Plasmids designed to alter the antibiotic resistance expressed by insertion mutations in *Bacillus subtili*s, through *in vivo* recombination. Gene. 1994 May 3;142(1):79-83.
6. Ishiwa H, Shibahara H. New shuttle vectors for *Escherichia coli* and *Bacillus subtilis*. II. Plasmid pHY300PLK, a multipurpose cloning vector with a polylinker, derived from pHY460. Jpn. J. Genet. 1985;60(3): 235-43.
